# Ranolazine: An Old Drug with Emerging Potential; Lessons from Pre-Clinical and Clinical Investigations for Possible Repositioning

**DOI:** 10.3390/ph15010031

**Published:** 2021-12-25

**Authors:** Sarah Rouhana, Anne Virsolvy, Nassim Fares, Sylvain Richard, Jérôme Thireau

**Affiliations:** 1 PhyMedExp, Université de Montpellier, Inserm U1046, CNRS UMR 9214, 34 295 Montpellier, France; sarah_rouhana89@hotmail.com (S.R.); anne.virsolvy@inserm.fr (A.V.); thireau.jerome@gmail.com (J.T.); 2 Research Laboratory in Physiology and Physiopathology, University Saint Joseph, Beyrouth 10999, Lebanon; nassim.fares@usj.edu.lb

**Keywords:** persistent Na^+^ current, hERG/IKr K^+^ current, arrhythmia, vascular, neuronal, metabolism

## Abstract

Ischemic heart disease is a significant public health problem with high mortality and morbidity. Extensive scientific investigations from basic sciences to clinics revealed multilevel alterations from metabolic imbalance, altered electrophysiology, and defective Ca^2+^/Na^+^ homeostasis leading to lethal arrhythmias. Despite the recent identification of numerous molecular targets with potential therapeutic interest, a pragmatic observation on the current pharmacological R&D output confirms the lack of new therapeutic offers to patients. By contrast, from recent trials, molecules initially developed for other fields of application have shown cardiovascular benefits, as illustrated with some anti-diabetic agents, regardless of the presence or absence of diabetes, emphasizing the clear advantage of “old” drug repositioning. Ranolazine is approved as an antianginal agent and has a favorable overall safety profile. This drug, developed initially as a metabolic modulator, was also identified as an inhibitor of the cardiac late Na^+^ current, although it also blocks other ionic currents, including the hERG/Ikr K^+^ current. The latter actions have been involved in this drug’s antiarrhythmic effects, both on supraventricular and ventricular arrhythmias (VA). However, despite initial enthusiasm and promising development in the cardiovascular field, ranolazine is only authorized as a second-line treatment in patients with chronic angina pectoris, notwithstanding its antiarrhythmic properties. A plausible reason for this is the apparent difficulty in linking the clinical benefits to the multiple molecular actions of this drug. Here, we review ranolazine’s experimental and clinical knowledge on cardiac metabolism and arrhythmias. We also highlight advances in understanding novel effects on neurons, the vascular system, skeletal muscles, blood sugar control, and cancer, which may open the way to reposition this “old” drug alone or in combination with other medications.

## 1. Introduction

**Figure 1:**
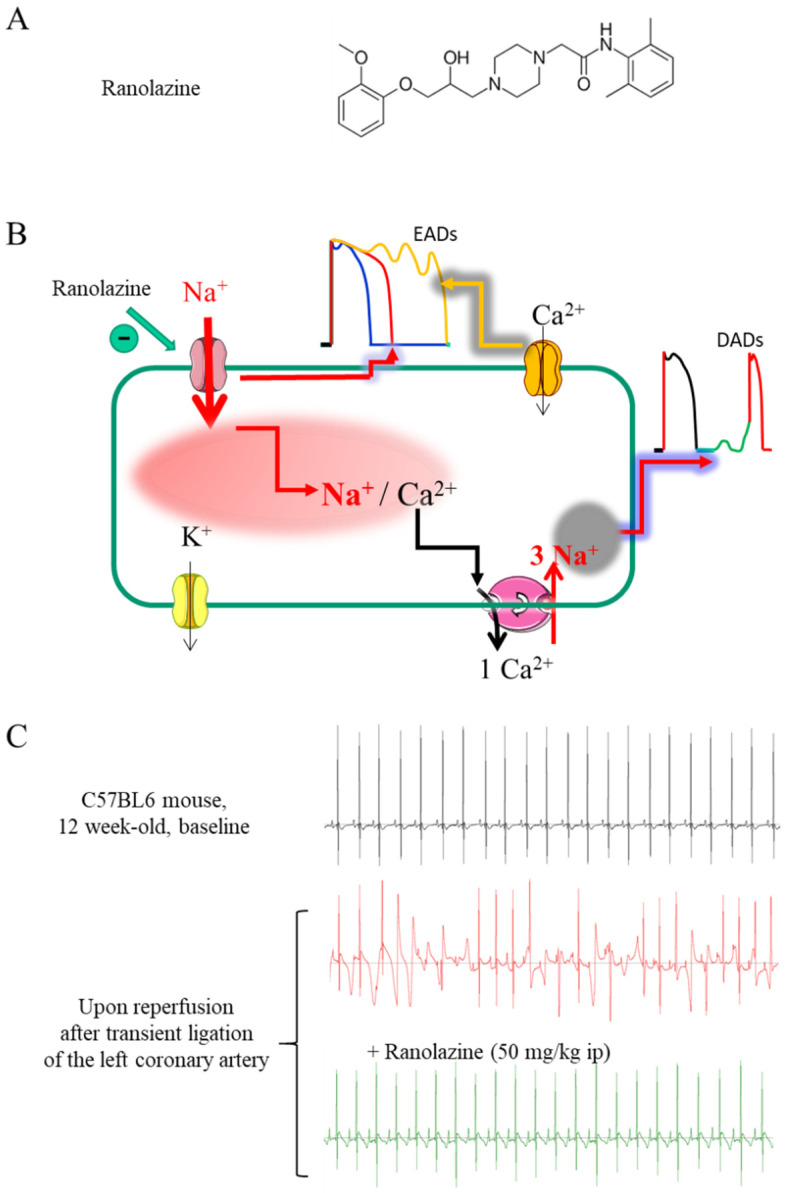
Benefits of I_Na,late_ inhibition by ranolazine on cellular cardiac action potential (AP) and Na^+^/Ca^2+^ ion homeostasis, and antiarrhythmic effects. (**A**) Chemical structure of Ranolazine. (**B**) Cellular impact of the sustained entry of Na^+^ caused by I_Na,late_ in cardiomyocytes. Two mechanisms cooperate: (**i**) the transmembrane influx of Na^+^, which maintains depolarization during the plateau phase of the action potential and can generate a re-activation of the Ca^2+^ channels responsible for early after depolarisations (EADs); (**ii**) the increase in intracellular Na^+^, which promotes an increase in intracellular Ca^2+^ and the occurrence of Ca^2+^-dependent delayed after depolarizations (DADs). Ranolazine prevents both mechanisms by shortening the AP and decreasing intracellular Na^+^ and Ca^2+^ in the presence of an I_Na,late_. (**C**) Typical electrocardiograms, recorded in 12-week-old male C57BL6 mice under gas anesthesia (Isoflurane 2.5%), during baseline conditions (**upper panel**) and after reperfusion following 25 min of ligation of the left coronary artery in the absence (middle panel) and in the presence of ranolazine (50 mg/kg i.p.) (**lower panel**) (unpublished personal data).

Ranolazine (Ranexa^®^) is a piperazine derivative [RS-43285; (6)-N (2,6-dimethyl-phenyl)-4[2-hydroxy-3(2-methoxy-phenoxy)propyl] 1-piperazine acetamide)] (Figure 1A) used as a second-line treatment in patients with stable or poorly controlled chronic angina pectoris and unresponsive to other drugs [1,2,3]. This non-hemodynamic anti-angina agent, patented in 1986 and approved by the Food and Drug Administration in 2006, is prescribed in the United States, Japan, and some European countries. However, ranolazine has other potential beneficial therapeutical effects in various cardiovascular pathologies, including post-operative, new-onset, paroxysmal, and chronic atrial fibrillation (AF), ventricular arrhythmias (VA), revascularization, coronary artery disease, diastolic and microvascular dysfunction, metabolic diseases, and diabetes [1,4]. A PubMed search by typing the word ‘ranolazine’ released more than 1100 references (1998–2021), showing the great interest this molecule has attracted due to its clinical potential and complex effects. Ranolazine indeed requires high doses to achieve an effect and could be considered a ‘dirty’ molecule. A low affinity for specific targets may account for the multiple outcomes and poorly understood action mechanisms. There is a trend in finding new uses for ranolazine (Table S1 in Supplementary Data), especially in diseases with unmet clinical needs. Recent data have shown that ranolazine can modulate the excitability of neurons in the peripheral nervous system, including the autonomic component, and the vascular system. The contribution of these effects to various potential benefits and limitations of ranolazine warrant further investigation. Here, we review the different clinical uses of ranolazine (Table S2 in Supplementary Data), its known mechanisms of action, and its potential for consideration in cardiac and other diseases.

## 2. Ranolazine as an Antianginal and Anti-Ischemic Drug

Ranolazine was first developed as an antianginal and anti-ischemic metabolic effector. Inhibiting fatty acid oxidation leads to a shift in myocardial energy production, from fatty acid oxidation to glucose oxidation, explaining ranolazine’s beneficial effects on cardiac metabolism and improved exercise tolerance without affecting heart rate or blood pressure [5,6,7]. The beneficial mechanism of the action of ranolazine in angina pectoris has been challenging to elucidate and has only recently begun to be clarified. It may involve indirect normalization of ion homeostasis. Indeed, during ischemia, changes in the ion homeostasis of cardiomyocytes are immediate. Alterations involve intracellular Na^+^, Ca^2+^, and H^+^ and extracellular K^+^ [8,9,10]. In particular, an increase in the amplitude of a persistent or late Na^+^ current (I_Na,late_), as found in several pathological situations, is sufficient to prolong the duration of the action potential (AP) and increase the intracellular concentration of Na^+^ [11]. Na^+^ extrusion through the Na^+^-K^+^ ATPase pump does not properly counterbalance the load of Na^+^, which increases intracellular Ca^2+^. The Ca^2+^ overload increases diastolic left ventricular (LV) pressure, causing a vicious cycle feedback loop on energy supply and demand, worsening angina pectoris. Indeed, the increase of intracellular Ca^2+^ in myocardial cells increases the tension of the diastolic wall and increases the end-diastolic pressure. The increase in the stiffness of the diastolic wall causes intra-myocardial vascular compression, which reduces blood flow and the supply of oxygen to the myocardium, then prevents ventricular filling [12]. In addition, an overload of Ca^2+^ can lead to cell damage and death if it is not corrected [13]. The increase in I_Na,late_ may be the main contributor to the phenomena observed in ischemia and hypoxia [14,15,16], paving the way for clinical trials showing therapeutic benefits of ranolazine [17].

Blocking of I_Na,late_ is the proposed mechanism in the proven efficacy and clinical indication for ranolazine treatment for stress angina. Blocking I_Na,late_ reduces intracellular Na^+^, Na^+^-induced Ca^2+^ overload and the subsequent deleterious effects on the diastolic pressure of the LV that exacerbate angina pectoris [2,3,18]. Effective concentrations have little or only minimal impacts on heart rate and blood pressure [19], leading to the hypothesis that ranolazine exerts its cardioprotective effects by a mode of action primarily separate from that of typical antianginal drugs such as Ca^2+^ channel antagonists, β-adrenergic receptor antagonists, and nitrates [17,20,21,22]. Experimental investigations showed that the Na_v_ channel agonist Anemone Toxin (ATX-II) mimics the effects of ischemia/reperfusion to increase intracellular concentrations of Na^+^ and Ca^2+^ [21]. Ranolazine could maintain coronary flow near normal levels during exposure to ATX-II. Therefore, besides its metabolic effect, ranolazine has been considered an I_Na,late_ blocker (i.e., up to 40-fold more specific for I_Na,late_ than I_Na,peak_ current, depending on models and conditions) [23]. Overall, these latter properties contributed to providing ranolazine with the status of an antiarrhythmic molecule (Table S1 in Supplementary Data).

## 3. Ranolazine as an Antiarrhythmic Drug

### 3.1. Ranolazine Has Multiple Ion Channel Effects

The electrophysiological effects of ranolazine were questioned early, mainly because of the worry of potential impacts on the QT interval in patients with severe chronic angina [17]. Ranolazine has multi-channel blocking effects, similar to those observed after chronic amiodarone, yet ranolazine has a much lower risk of Torsades de Pointes (TdP) than amiodarone [18,24,25]. Ranolazine inhibits the delayed rectifying potassium current (I_Kr_) (Kv11.1 channel encoded by the *KCNH2* gene or *HERG*) and the L-type calcium channel current (I_Ca,L_) at concentrations slightly higher than those required to inhibit peak Na channel current (I_Na,peak_), and has a modest and self-limited effect on the QT interval [26]. A comparative electrophysiological investigation performed in isolated canine LV myocytes indicated that ranolazine inhibits different currents with the following IC_50_: 11.5 µM for I_Kr_, 5.9 µM for I_Na,late_, 50 µM for late I_Ca,L_, 296 µM for peak I_Ca,L_, and 91 µM for I_NCX_ (NCX for Na^+^/Ca^2+^ exchanger) while they found no effect on I_to_ and I_K1_ [18].

Despite multiple molecular targets, the vast majority of clinical studies have reported good safety and tolerability of ranolazine, yet with some contraindications., mainly dizziness, headache, nausea, debility, constipation, and a variety of side effects that are not specific and most often shared by other drugs [4,27,28,29,30,31,32,33]. Hence, many clinical trials found that short- and long-term treatments with ranolazine are safe despite a modest QT lengthening effect [23,28,29,34]. Administration in patients with a family history of long QT syndrome or known prolonged QT interval may be contra-indicated. The relative safety of ranolazine may relate to its ability to decrease I_Ca,L_ at concentrations close to those inhibiting I_Kr_ in addition to I_Na, late,_ thus producing offsetting effects on repolarization at doses above 20 µM [26,35,36]. Ranolazine’s multiple ion channel effects may support its “self-limitation” to trigger TdP [18].

As ranolazine does not trigger TdP despite its QT lengthening effect, clinical studies investigated its antiarrhythmic efficacy because of its interesting electrophysiological effects, including a significant blocking impact on I_Na,late_. Ranolazine could suppress early afterdepolarizations (EADs) and reduce transmural dispersion of repolarization, suggesting antiarrhythmic activity [18]. Conversely, by normalizing Ca^2+^ homeostasis and repolarization heterogeneity, ranolazine could prevent malign VAs in the dog model of chronic atrioventricular block [37]. This trend was initially observed in several experimental models of long QT syndrome (LQT1, LQT2, and LQT3) [38,39,40] and then in patients with LQT3 in whom ranolazine (starting from 1 mg/mL) shortened QTc without modifying the conduction time (PR and QRS) [41]. The benefit of I_Na,late_ blocking effect by ranolazine should not be reduced to those pathologies involving specific mutations in Na_v_ isoforms [42,43], since I_Na,late_ also develops in several pathological and pharmacological cardiovascular conditions, including bradycardia, myocardial ischemia, reperfusion injury, and heart failure (HF) with nefast consequences on morbidity and mortality [22].

### 3.2. Clinical Evidence of Antiarrhythmic Benefits

The MERLIN-TIMI 36 trial was the first study to assess ranolazine’s antiarrhythmic effect (documented by electrocardiogram, ECG) in 6345 patients with non-ST-segment elevation acute coronary syndrome and moderate to high risk for death or recurrent ischemic events [44]. The addition of ranolazine to standard treatment was ineffective in reducing major cardiovascular events. However, ranolazine significantly suppressed supraventricular tachyarrhythmia and reduced new-onset AF by 30%. In addition, subsequent analysis showed that the overall burden among patients with paroxysmal AF tended to be lower with ranolazine than with placebo, with fewer AF-related adverse events [45]. Indeed, overall, patients assigned to ranolazine had a trend towards fewer episodes of AF (75 (2.4%) vs. 55 (1.7%) patients, *p* = 0.08) from ECG during the seven first days after randomization. The pattern of new-onset AF differed between the ranolazine vs. placebo group: clinically insignificant AF (five patients in ranolazine vs. seven in placebo), paroxysmal AF (18 vs. 48 patients), and predominantly chronic AF (28 vs. 20 patients, three-way *p* < 0.01). Among patients with a paroxysmal AF pattern, the overall burden was lower with ranolazine than with placebo (median 4.4 vs.16.1%, *p* = 0.015). Over the median 1-year follow-up, fewer patients treated with ranolazine experienced an AF event than placebo (2.9 vs. 4.1%, RR 0.71, *p* = 0.01). Many studies confirmed this trend with ranolazine used alone [46] or in combination with amiodarone/dronedarone [47,48,49] or ivabradine [50]. Short treatment with ranolazine also prevents AF following coronary artery bypass graft and valve surgery [51] and is more effective than amiodarone in preventing AF following coronary artery bypass surgery. Ranolazine can also be used for acute termination of new or paroxysmal AF as a “pill in the pocket” drug. In the RAFFAELLO trial, ranolazine prolonged the time to AF recurrence after successful electrical cardioversion and reduced AF recurrences [52].

Compared with the abundant data on ranolazine utility and efficacy in AF of different etiologies, only a few clinical studies investigated its use in ventricular rhythm disturbances. In the MERLIN-TIMI 36 trial, ranolazine reduced non-sustained ventricular tachycardia (VT) (i.e., at least eight successive abnormal ventricular complexes). The incidence of supraventricular arrhythmias was also reduced [44]. More recently, in a small cohort of patients with documented symptomatic premature ventricular contraction, ranolazine successfully reduced ventricular bigeminy by 80% (*p* < 0.001), ventricular couplets by 78% (*p* < 0.001), and finally VT by 91% (*p* < 0.001) in a dose-dependent manner, underlying the need for a large, prospective randomized study [31].

In patients with drug-refractory shocks delivered by implantable cardioverter-defibrillator (ICD), ranolazine reduced VT burden and ICD shocks in 11/12 patients [53]. By contrast, a recent study in patients with ICD showed that ranolazine did not significantly reduce the incidence of VT, ventricular fibrillation (VF), or death in high-risk patients. However, in the last, the investigators highlighted that this study was underpowered to detect a difference in the primary endpoint (death). Nevertheless, in pre-specified secondary endpoint analyses, ranolazine administration was associated with a significant reduction in recurrent VT or VF requiring ICD therapy, without evidence for increased mortality [54]. In parallel with clinical evidence supporting antiarrhythmic effects (Table S2 in Supplementary Data), the mode(s) of action of ranolazine has been further explored in pre-clinics from biophysics to molecular, cellular, and in vivo electrophysiology to attempt to reveal its secrets.

### 3.3. Antiarrhythmic Mechanisms at the Ventricular Level

Antzelevitch’s group revealed significant antiarrhythmic effects and the main targets of ranolazine. This drug exhibits antiarrhythmic action within its therapeutic range as an antianginal agent (i.e., 2–6 µM) [18]. Tonic blockade of I_Na,late_ is central at the ventricular level, while use-dependent inhibition of peak I_Na_ and I_Kr_ may underlie the atrial effects. It is worth noting that although a block of the peak I_Na,late_ is more robust in the atria than the ventricles in dogs, fast pacing rates and depolarized resting membrane potential can induce substantial inhibition of I_Na_, both peak and late, with class I/B antiarrhythmic characteristics in ventricular preparations [35,55].

In normal conditions, cardiac voltage-gated Na^+^ channels (Na_v_) open quickly and transiently to support the fast AP upstroke and then inactivate relatively quickly (milliseconds scale). Among the different Na_v_ isoforms (from 1.1 to 1.8), Na_v_1.5 encoded by the *SCN5A* gene is the main Na_v_ channel in the heart [56,57]. This isoform generates the cardiac I_Na,late_, yet Na_v_1.8, encoded by the *SCN10A* gene, may also be involved in cardiac conduction and occurrence of VA [58,59,60,61]. A tiny I_Na_ fraction (less than 0.1–1% of the global peak I_Na_ amplitude) can persist in some pathophysiological situations and is referred to as I_Na,late_ [62,63,64]. I_Na,late_ can last for several hundreds of milliseconds after the peak of I_Na_. The evidence of a role for I_Na,late_ in arrhythmogenesis was first derived from experiments on rat ventricular myocytes in hypoxic conditions [14,65,66]. This property, thus, provides ranolazine the status of a potential antiarrhythmic molecule as illustrated in (Figure 1C). Indeed, I_Na,late_ promotes abnormal automaticity and arrhythmia via at least two distinct mechanisms.

The first pro-arrhythmogenic function of I_Na,late_ occurs at the cardiomyocyte’s membrane compartment level, promoting prolonged depolarization throughout the cardiac AP plateau and providing a substrate for EADs [67]. The AP lengthening lengthens the QT interval of the ECG, initiates and perpetuates re-entry mechanisms until VT and TdP [68,69]. Typically, mutations in the *SCN5A,* associated with type 3 prolonged QT (LQT3) syndrome, slow inactivation of Na_v_ channels and promote I_Na,late_ [70]. A study in HEK cells expressing the Na^+^ channel R1623Q mutation responsible for LQT3 syndrome showed I_Na,late_ inhibition at low ranolazine concentrations [55]. In long QT syndromes (LQT1, LQT2, and LQT3), ranolazine efficiently suppresses TdP by normalizing repolarization stability and dispersion in several experimental models [23]. Ranolazine also efficiently reduces TdP triggering by xenobiotics, promoting the *HERG* blocking effect [37,38,71,72,73].

Ranolazine significantly reduces AP duration and repolarization instability in ventricular cells. However, it has no significant effects on the resting membrane potential in different models (canine ventricular cardiomyocytes, guinea pig papillary muscles and ventricular myocyte, canine atrial tissue, and Purkinje fibers) [18,74,75]. Ranolazine probably does not affect I_K1_ in ventricular and atrial cardiomyocytes [18,75]. Ranolazine-mediated AP duration decrease in ventricular cells is primarily due to significant inhibition of I_Na,late_ current, and I_Kr_, yet at different doses (IC_50_~6 µM for late I_Na_ and 12 µM for I_kr_) [18,76]. Depending on the cell type, the regional expression heterogeneity and the respective contribution of these currents on AP duration is a significant ranolazine asset. In cells with high I_Na,late_ expression, and long AP duration (e.g., Purkinje and M cells), ranolazine shortens AP duration more efficiently, dose- and frequency-dependently [77,78,79]. Reduced outward conductance such as I_Kr_ currents favors this effect [71]. It is especially true when the AP duration is pharmacologically lengthened by *HERG* blocking (human Ether-à-go-go-Related Gene) channel drugs by blockers of Na^+^ channel inactivation, such as the ATX II, or during angiotensin treatment. In these conditions, ranolazine drastically shortens AP duration and reduces the dispersion of ventricular repolarization and beat-to-beat variability of AP duration [18,37,76,80,81,82], and reduces risk factors for promotion of EADs and cardiac death following arrhythmias [83,84].

The second pro-arrhythmogenic impact of I_Na,late_ involves intracellular Na^+^ accumulation in the cytoplasm (Figure 1B). The rise in cytoplasmic Na^+^ fuels intracellular Ca^2+^ overload via the Na^+^/Ca^2+^ exchanger (NCX) activity. Spontaneous sarcoplasmic reticulum (SR) Ca^2+^ release (Ca^2+^ sparks) through the ryanodine receptor RyR2 can thus generate cytoplasmic Ca^2+^ waves as seen, for example, in HF or catecholaminergic polymorphic ventricular tachycardia [85]. Likewise, Ca^2+^ leakage from the SR is also involved in atrial arrhythmia (as AF) [86]. In these conditions, the NCX activated by the Ca^2+^ waves produces an electrogenic depolarizing Na^+^ current (I_ti_) that enables firing of I_Na_, spontaneous AP, delayed after-depolarizations (DADs), and triggered activity [11,87]. Such phenomena observed in failing ventricular myocytes of different species [88,89] are normalized by ranolazine, which can decrease diastolic Ca^2+^ accumulation, to prevent the electrophysiological consequences of Ca^2+^ leak from the SR and finally to avoid the triggering of abnormal spontaneous Ca^2+^ waves in ventricular myocytes during ischemia or after ATX-II dosing [21,90,91].

### 3.4. Antiarrhythmic Mechanisms at the Atrial Level

In addition to beneficial effects on VA, ranolazine can effectively treat AF, maintaining normal sinus rhythm in AF patients [44,46,92]. Several experimental model studies showed that the drug prevents atrial ectopic beats and AF initiation. Ranolazine may predominantly delay the atrial rather than the LV AP, accounting for the more significant benefits in AF and lower risk for ventricular side effects [75,92,93]. The antiarrhythmic action involves inhibiting different ion currents such as the I_Kr_, fast inactivating I_Na_, and I_Na,late,_ [55,64]. The consequence is lengthening the atrial AP duration and effective refractory period (ERP) in a use-dependent manner, thus reducing excitability. Of note, ranolazine also inhibits TASK-1, an atrial-specific two-pore domain K^+^ (K2P) channel upregulated in AF [94].

Ranolazine has a putative atrial-selective action against voltage-gated Na^+^ channels [75], which may account for differences in the biophysical properties of I_Na_ as seen between rabbits atrial and ventricular myocytes [95]. Ranolazine potently inhibits I_Na,peak_ in atrial cells, but not in ventricular cardiomyocytes (weak effect) [75,96,97]. Indeed, I_Na,peak_ current inhibition also depends on the membrane potential and is more significant in depolarized myocytes, such as atrial myocytes. The activation and steady-state inactivation of atrial I_Na_ at more negative voltages, together with the higher state-dependent affinity of the drug for inactivated Na_v_ channels, may also underly the atrial-selectivity of ranolazine [95]. In addition, chronic AF promotes atrial I_Na,late_ [96], which is likely to reduce the AP firing threshold, initiate depolarization, and increase excitability and atrial arrhythmias turning into AF [98,99]. As in ventricular myocytes, I_Na,late_ can induce DADs [99] through a mechanism involving SR Ca^2+^ leak and calcium/calmodulin-dependent protein kinase II (CaMKII) activation [100,101].

This mechanism prolongs atrial AP duration in AF associated with diseases such as congestive HF [100], long QT syndrome [102], or atrial remodeling [103]. Similarly, AF may occur in ischemia/reperfusion, after cardiac surgery, and during hypertrophy/HF [104]. Ranolazine prevents the induction of β-adrenergically-mediated AF and avoids or suppresses persistent AF mediated by vagal stimulation in coronary-perfused canine atria in ischemia/reperfusion conditions [75]. Ranolazine showed similar inhibitory effects on induction or duration of AF initiated by vagal stimulation in intact porcine [105] and canine hearts [106] and a rabbit model of inducible atrial tachyarrhythmia elicited by acetylcholine [107].

Reactive oxygen species (ROS) overproduction and oxidative stress promote AF in multiple pathologies, such as in reperfusion and even during aging, promoting EADs and DADs [108]. Ranolazine normalizes repolarization, suppresses H_2_O_2_-induced EADs and DADs elicited through I_Na,late_ current modulation in isolated atrial guinea pig myocytes [99,109]. Ranolazine also terminates induced-atrial flutter and AF in the canine sterile pericarditis model by prolonging the ERP [110]. Similarly, in canine pulmonary vein sleeves, ranolazine causes marked use-dependent inhibition of Na^+^ channel activity, leading to lengthening the ERP, conduction slowing, and blocking and suppressing late phase 3 EADs and DAD-mediated triggered activity [111]. Furthermore, low concentrations of ranolazine or dronedarone produce weak electrophysiological effects and AF suppression when used independently. Conversely, they exert potent synergistic effects when combined, resulting in atrial-selective depression of Na^+^ channel-dependent parameters and effective AF suppression [112]. This work led to a clinical trial showing that in combination, ranolazine (moderate dose) and dronedarone (reduced dose) synergistically reduce AF burden with good tolerance/safety [48]. Similarly, recent experimental work in horses has shown that, compared to single drugs, the combination of dofetilide and ranolazine increased the antiarrhythmic effects on acutely induced AF, affecting cardioversion time, vulnerability at AF, and the duration of AF [113].

## 4. Non-Cardiac Effects of Ranolazine

### 4.1. Neuronal Effects

Ranolazine targets Na_v_ channel persistent activity, i.e., when Na_v_ channels fail to inactivate after opening or eventually re-opening [114,115]. Ranolazine can interact with a broad spectrum of Na_v_ channel isoforms, including neuronal isoforms, which opens exciting perspectives, yet it may not be ideal for specific clinical purposes [115]. For example, ranolazine can block I_Na,late_ evoked by mutations of *SCN1A*, the gene encoding the pore-forming subunit of the Na_v_1.1 channel isoform, frequently involved in altered neuronal excitability associated with a spectrum of genetic epilepsies and a familial form of migraine [116]. Accordingly, neuronal effects of ranolazine have been reported in vivo, opening the possibility for therapeutic applications in the treatment of central neuronal disorders, including inherited forms of epilepsy and a familial form of migraine associated with a persistent Na^+^ current due to slow inactivation or repetitive firing with exaggerated Na^+^ channel opening [116,117].

A clinical trial is underway to test whether ranolazine reduces neuronal hyper-excitability, slows disease progression, and reduces cramp frequency in amyotrophic lateral sclerosis, but the report is currently pending (NCT03472950, University of Kansas Medical Center). The protective mechanism against neuronal ischemia is similar to that reported for riluzole by blocking the I_Na,late_ in several models [118,119,120]. Another selective I_Na,late_ blocker, GS967, has also displayed potent antiepileptic activity [121]. In addition to inhibition of I_Na,late_ in cardiac cells and antiarrhythmic effects [122,123], GS967 inhibits I_Na,late_ and spontaneous AP firing in pyramidal neurons and prevents both hilar neuron loss and development of mossy fiber sprouting, suppresses seizure activity, and improves survival in genetically epileptic *SCN2A*Q54 mice [121].

**Figure 2:**
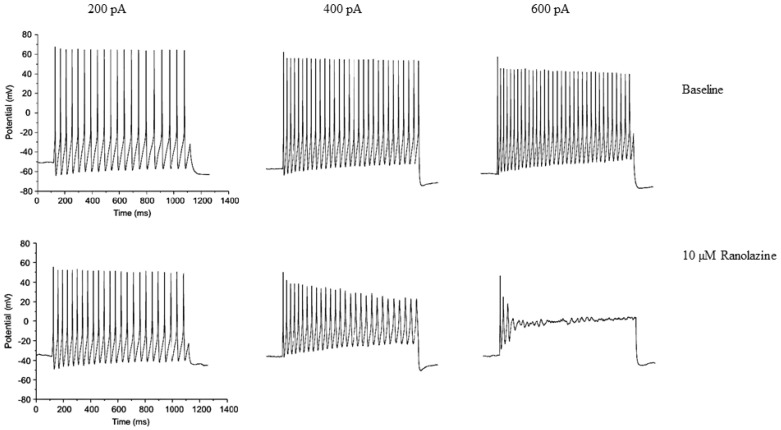
Clinically relevant concentration of ranolazine blocks high-frequency firing of Dorsal Root Ganglion (DRG) neurons expressing the wild-type (WT) Nav1.7 isoform. Traces illustrating the response (high-frequency firing phenotype) to 1-s duration current injections of 200 pA, 400 pA, and 600 pA both before (**upper row**) and after (**lower row**) exposure to 10 μM ranolazine in DRG neuron transfected with human Na_v_1.7-WT channels. Reproduced from Figure 6B [125], an open-access article distributed under the terms of the Creative Commons CC-BY 2.0 Attribution license.

Neuronal effects of ranolazine and other I_Na,late_ blockers open the perspective of an impact on peripheral neurons, excitability, and, indirectly, on the cardiovascular system. The importance of such effects in heart-brain communication may be worth considering for future therapeutic strategies [124]. Ranolazine may normalize the activity of the “neuronal” I_Na,late_ responsible for increasing the excitability of dorsal root ganglion neurons (DRG) overexpressing Na_v_1.7 (Figure 2). These neurons are involved in neuropathic pain associated with peripheral nerve excitability [125,126]. Ranolazine also improves behavioral signs of neuropathic pain associated with Na_v_1.7 and Na_v_1.8 isoforms [127]. Because I_Na,late_ currents, especially at or near the threshold for AP firing, can increase excitability, drugs that selectively target these currents in nociceptive neurons could be useful in treating pain [115]. In neurons, as in cardiac cells, the effect of ranolazine is state-dependent, as shown for Na_v_1.1, Na_v_1.2, and Na_v_1.7 [117,128]. Ranolazine may interact with the Na_v_ channels inactivated states, reducing excitability and epileptiform activity in neuronal cultures [116,117,125,128].

The neuronal isoforms Na_v_1.1 and Na_v_1.6 increase proportionally with I_Na,late_ in pressure-overloaded rat hearts [129,130,131]. Of significant interest, ranolazine preserved or improved LV ejection fraction during a 24 month follow-up period when added to guideline-driven therapy in congestive HF [30,132]. The high sympathovagal balance was improved, which directly affected autonomic Na^+^ channels. In line with clinical data, ranolazine delivered intraperitoneally in rats with chronic HF alleviated sympathetic nerve activity and improved the impaired LV function, amplified following vagal activation [133]. Consistently, ranolazine attenuated the heightened norepinephrine and B-type natriuretic peptide-45 and improved cardiac function in rats with chronic ischemic HF [134]. In addition to electrophysiological actions, neuroprotection and cardioprotection may involve anti-inflammatory and antioxidant effects [135,136,137,138,139]. Future studies with ranolazine may consider these benefits due to the recent success of non-specific anti-inflammatory molecules after myocardial infarction [140].

### 4.2. Vascular Effects of Ranolazine

**Figure 3:**
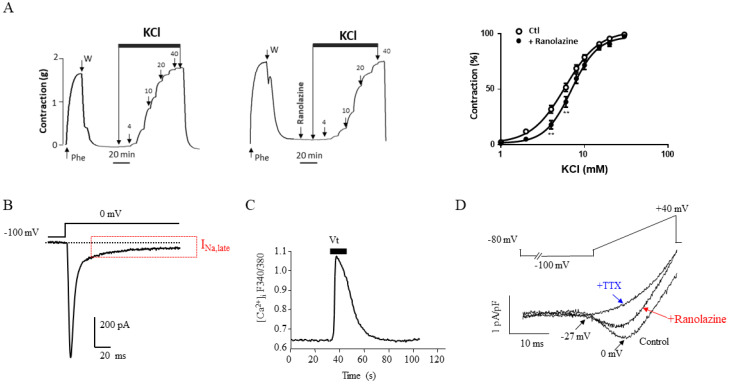
Voltage-gated Na^+^ currents in arteries and effect of ranolazine. Effect of the addition of cumulative doses of KCl (1 to 40 mM) on a Na_v_ channel-dependent component of contraction recorded in the absence (**left**) and the presence of ranolazine (20 μM) (**right**) in rat aortic rings. Graphs summarize the dose-response curves obtained for KCl. Averaged data expressed as percentage of the maximal contraction induced by KCl (*n* = 15) (**A**). Reproduced from Figure 3B in [142]. (**B**) Typical Na^+^ current with an I_Na,late_ evoked at a test potential of 0 mV from a holding potential of −100 mV, using the whole-cell patch-clamp technique, in a primary cultured human coronary myocyte (HCM) (unpublished personal data). The experiment was performed as described [161]. (**C**) Increasing effect of the Na^+^ channel agonist veratridine (Vt; 10 μM) on intracellular Ca^2+^ ([Ca^2+^]_i_) in a fura-2–loaded HCM (unpublished personal data). The experiment was performed as described in [162]. (**D**) Antagonist effect of ranolazine (20 µM) on a veratridine-induced I_Na_ in a primary cultured rat aortic myocyte. The I_Na_ current was evoked by a 40 ms ramp from −80 mV to +40 mV, following a 2-sec prepulse at −100 mV, from a holding potential of −80 mV in the presence of Vt (100 μM). Tetrodotoxin (TTX; 1 µM) was added after ranolazine to block all I_Na_ current. Arrows indicate the current’s activation and maximal amplitude with the corresponding voltages. Reproduced from Figure 1A in [142].

The primary mechanism of ranolazine antianginal benefits was first established and emphasized as cardiac protection against the metabolic consequences of ischemia. This mechanism contrasts markedly with other classical antianginal drugs that directly affect coronary flow. However, there is now strong experimental and clinical evidence of ranolazine vascular effects, which may contribute to its beneficial effects in patients with stable angina [141]. Indeed, ranolazine induces dose-dependent relaxation of arterial rings from healthy (Figure 3A) and diabetic rats, previously contracted with phenylephrine [141,142,143], and substantially improves the regional coronary blood flow in areas of myocardial ischemia [144]. At therapeutic doses, ranolazine also exerts an additive vasorelaxant effect in rabbit aortic rings when combined with nicardipine [145]. Although other reports challenge this effect [143], studies confirmed that ranolazine could improve angina and myocardial perfusion in patients with severe coronary microvascular dysfunction [146,147,148]. Specifically, the synthesis/release of nitric oxide by the endothelium might also contribute, yet the ranolazine vasodilatory effect is predominantly endothelium-independent [141,142,149,150]. Ranolazine inhibits Na_v_ channels and antagonizes α_1_-adrenergic receptors in vascular smooth muscle cells (VSMCs), in line with its pleiotropic effects [142,151]. Blockade of α_1_-adrenergic receptors may also account for vasodilatory effect after intracoronary or intra-femoral bolus injection in anesthetized pigs [152]. Experiments in rat intrarenal arteries reached similar conclusions [153]. Ranolazine could also improve vasodilatation through relaxant and antiadrenergic effects in the human saphenous vein [154]. Several groups recently reported potential interest in ranolazine in pulmonary arterial hypertension by reducing cardiovascular death in an experimental rat model. Thus, ranolazine may improve pulmonary hemodynamics, alleviate cardiac remodeling (right ventricle) and improve susceptibility to ventricular arrhythmia [155,156]. Ranolazine was also reported as a safe treatment in preliminary works on pulmonary arterial hypertension [157,158], which was confirmed recently in a clinical trial [159,160].

Suggesting vascular channel inhibition by ranolazine was provocative and somehow unexpected because its antianginal benefits were primarily attributed to its effects on cardiac I_Na,late_. The notion of vascular I_Na_ is more recent than that of neuronal, cardiac, and muscular I_Na_ present in excitable cells [163]. Vascular I_Na_ currents have been identified in rabbit and human pulmonary arteries and various primary VSMCs from the arterial smooth muscle layer (rat, pig, and human) [161,164,165,166,167,168]. Notably, we found an atypical I_Na_ in primary cultured human coronary and aortic myocytes (Figure 3B). This I_Na_ is activated at more positive potentials than most I_Na_ subtypes and exhibits a large late component that is related to inactivation failure and is observed even for large depolarizations (>0 mV) [161,162,166]. This current causes a basal Na^+^ influx into myocytes that regulates both intracellular sodium ([Na^+^]_i_) and Ca^2+^ ([Ca^2+^]_i_) via activation of depolarization-gated Ca^2+^ channels and NCX [162]. The beneficial effects observed by ranolazine, mentioned above, may result from an inhibition of the pulmonary vascular I_Na,late_ to explain its effect in pulmonary hypertension [169], in line with the presence and blockade of TTX-sensitive Na_v_ channel isoforms in human pulmonary artery VSMCs [168,170]. Veratridine induces Na_v_ channel-dependent increases in intracellular Ca^2+^ primary cultured human coronary myocytes (Figure 3C), and the I_Na_ promoted by veratridine can be blocked by ranolazine in rat primary cultured aortic VSMCs (Figure 3D). These effects could contribute to the benefits of ranolazine in patients with stable ischemic heart disease compared with traditional antianginals, such as beta-blockers, Ca^2+^ channel blockers, or long-acting nitrate [171].

### 4.3. Gluco-Metabolic Effects of Ranolazine

A promising therapeutic approach for ischemic heart disease and HF is metabolic modulation to optimize energy substrate utilization [172]. Ranolazine may preserve the LV ejection fraction and decrease high sympathovagal balance when added to guideline-driven therapy in chronic HF [132]. Several animal studies and at least three double-blinded, randomized, placebo-controlled clinical trials have brought data in favor of a beneficial effect of ranolazine as a metabolic modulator [173]. Ranolazine is efficient in short-term or intermittent ischemia conditions and during stress challenge or exercise but presents no beneficial effect in “no-flow ischemia” situations [173]. Ranolazine stimulates glucose oxidation and partially reduces fatty acid oxidation, leading to improved ATP production/O_2_ consumption ratio, and diminished H^+^, lactate, and harmful fatty acyl intermediates [174]. Ranolazine may promote the utilization of substrates to produce enough energy to improve contractile performance. In an isolated rabbit heart model, ranolazine consistently limited the decrease in cardiac ATP during ischemia in a concentration-dependent manner, allowing a significant cardio-protective effect during ischemia and reperfusion [175].

Recent studies have re-emphasized that ranolazine exerts a well-tolerated glucometabolic effect and positive glucose control in patients with diabetes [4,32,173]. In particular, a meta-analysis showed improved glycated hemoglobin (HbA1c) without increased risk of hypoglycemia, thus benefiting patients with type 2 diabetes and chronic stable angina [176,177,178]. Ranolazine was also proposed as first-line therapy in diabetes and coronary artery disease [179,180]. Thus, ranolazine may complement or supplant traditional drugs, especially those with potentially harmful hemodynamic effects [178]. Interestingly, during exercise, patients receiving ranolazine generate more cardiac work than those receiving placebo. Unlike atenolol, ranolazine’s antianginal and anti-ischemic effects did not depend on decreased cardiac work affected by reductions in heart rate, blood pressure, or rate–pressure product [181]. Moreover, patients treated with ranolazine can exercise for a longer time before the appearance of angina symptoms and myocardial ischemia (ST-segment depression) [182,183].

### 4.4. Skeletal Muscle Effects of Ranolazine

Patients with myotonia congenita present muscle hyperexcitability due to loss-of-function mutations in the ClC-1 chloride channel in skeletal muscle. These mutations cause involuntary firing of muscle action potentials (myotonia), producing muscle stiffness due to slow afterdepolarization. The individuals experience spells of muscle stiffness or when the muscles do not relax after contracting. The mechanism involved a late Na^+^ current triggering spontaneous myotonic AP. Patch-clamp studies on muscle from a mouse model of myotonia congenita allowed to conclude that the ideal myotonia therapy would selectively block a sustained Na^+^ current induced by loss-of-function mutations in the ClC-1 chloride channel and spare the transient Na^+^ current [184,185]. Ranolazine was thus shown efficient in this pathology [184]. Another group obtained similar results and noted that ranolazine produced fewer side effects and was as effective as mexiletine at a dose that had none of the mexiletine’s hypoexcitability side effects [186]. Following these observations, a pilot study on thirteen participants established that ranolazine could improve signs and symptoms of myotonia and muscle stiffness in patients with myotonia congenita. In this study, ranolazine appeared to be well tolerated over four weeks and improved signs and symptoms of muscle stiffness. The findings of this study suggested investigating ranolazine’s effect in a more extensive controlled study [187,188]. Similarly, a study indicated that a block of human Nav 1.4 is helpful to reduce the sustained AP firing in paramyotonia congenita [189]. This work paved the way for a single-center trial of ranolazine to evaluate efficacy and tolerability in patients with paramyotonia congenita. In this study, the subjective symptoms of stiffness, weakness, and pain, as well as clinical and electrical myotonia, were evaluated. This study supported the use of ranolazine as a treatment for myotonia in paramyotonia congenita and suggested that a randomized, placebo-controlled trial is warranted [187].

### 4.5. Ranolazine and Cancer

The capacity to metastasize is one of the hallmarks of cancer, and usually, death due to cancer is not caused by the primary tumor but rather by the metastatic spread. The lack of effective therapy for preventing metastasis results in a high mortality rate in oncology. Reducing the risk of metastasis may significantly improve survival and quality of life [190]. Old drugs and compounds have shown anti-metastasis activity by acting on the invasive capacity of these cells [190]. Cancer cells expressed functional voltage-gated Na channels playing a significant role in disease progression in the prostate, breast, lung cancers, and leukemia [191]. Thus, the expression of Na_v_ channels in tumor cells questions their role in cancer therapy and opens perspectives for a potential new target in oncology [191,192,193]. Indeed, Na_v_1.7, Na_v_1.6, and Na_v_1.5’s functional expression are associated with invasive properties of some cancer cell lines [191]. In cancer cells, Na_v_ channels are not involved in AP genesis as in excitable cells and instead serve to regulate resting Ca^2+^ homeostasis, essential proteases release, and pH regulation through Na^+^-H^+^ exchanger, two phenomena important to cancer invasiveness. In addition, cancer cell lines that express Na_v_ are more metastatic and correlate with patient mortality [194]. Thus, channel blockers were tested in cancer, particularly blockers of I_Na,late_ [195,196] such as ranolazine assessed on breast cancer cell invasiveness and lung colonization [197]. In vitro, ranolazine inhibits Na_v_ currents and reduces invasiveness in breast cancer cells. In vivo, the injection of ranolazine significantly reduced lung colonization by human breast cancer cells in immunodepressed mice with no apparent toxic effect. Similar results were recently obtained in a rat model of prostate cancer [198]. In addition to its electrophysiologic effects, the metabolic modulatory effect of ranolazine was also tested in cancer. Targeting fat oxidation in mouse prostate cancer decreases tumor growth and stimulates anti-cancer immunity [199]. By contrast, by favoring ATP production, Suckow et al. observed that ranolazine caused a dose-dependent increase in tumor number in APC(Min/+) mice, a model of spontaneous intestinal tumorigenesis [200]. Furthermore, in an in vitro study of colorectal cancer invasiveness, ranolazine increased invasiveness under hypoxia, whereas its effect was lower under normoxia [201]. To our knowledge, the efficacy and safety of ranolazine or other Na_v_ channel blockers in oncology have not been assessed in clinical trials. However, ranolazine may reduce the cardiotoxicity of anticancer therapy (trastuzumab, doxorubicin) in mice, thus paving the way to a clinical trial [138,202,203,204]. A controlled trial is needed regarding the potential of ranolazine on relieving chemotherapy-related diastolic dysfunction and its safety profile in cancer patients compared to that of the general population [204].

## 5. Adverse Effects of Ranolazine

Numerous trials assessed ranolazine tolerability and safety. Ranolazine is beneficial in angina as monotherapy (MARISA study) and in combination with other suboptimal antianginal agents (CARISA, RAN080). Long-term therapy seems well-tolerated without significant clinically hemodynamic effects in patients with chronic angina [20]. However, clinical studies have reported adverse reactions [205]. Overall, 6% of patients discontinued treatment due to an adverse event (vs. 3% in the placebo groups), mainly dizziness, headache, nausea, debility, and constipation (https://www.ncbi.nlm.nih.gov/books/NBK507828/ accessed on: 10 December 2021). Syncope, confusion, tinnitus, vertigo, blurred vision, dyspnea, hematuria, bradycardia, palpitations, hypotension, orthostatic hypotension, thrombocytopenia, leukopenia, abdominal pain, dry mouth, vomiting, anorexia, dyspepsia, peripheral edema, angioedema, renal failure, eosinophilia, paresthesia, tremor, pulmonary fibrosis, and excessive sweating have also been reported. For example, during the first two years of the Ranolazine Open Label Experience (ROLE) study, the occurrence of an adverse effect was the most common reason for treatment discontinuation in 10% of patients [28]. In this study, adverse events were mainly general (dizziness) and digestive (constipation) and concerned nearly 12% of patients enrolled in the study [28]. In the MERLIN TIMI randomized trial (*n* = 6560 patients included within 48 h of an acute coronary syndrome), the ranolazine tolerance profile was favorable in patients with prior angina [29]. The most common adverse effects were similar to those recorded in the ROLE study: dizziness (12%), nausea (10%), and constipation (9%) [29]. Overall, ranolazine was discontinued due to an adverse event by 8% of patients [29]. The reported side effects were not specific as they are shared by other Class I [206] and some Class III anti-arrhythmic drugs [207,208].

Ranolazine side effects are strongly related to the administered dose and the presence of hepatic and/or renal impairment. For example, in the MERLIN TIMI trial, ranolazine dose (1000 mg twice per day per os) was decreased in 11% of patients due to renal dysfunction, in 0.6% of patients due to persistent prolongation of the corrected QT interval, and in 8.6% of patients due to other adverse events [29]. In the MARISA trial (500 mg, 1000 mg, and 1500 mg twice per day), study interruption for adverse events was more frequent in the 1500 mg group [20]. Another study described the first case of neurologic adverse events in an 81-year-old woman with coronary artery disease, renal impairment, and mild neurologic disease who received ranolazine for symptomatic control of a non–ST-segment elevation myocardial infarction [209]. The patient started with 500 mg twice per day at admission, and on day 3, the dose was increased to 1000 mg twice per day. Just 48 h after the dose increase, she experienced dysarthria, dysmetria, hallucinations, worsening of tremors, and difficulty in word-finding, probably due to the combined effect of the dose increase and favoring factors, like advanced age, renal impairment, and baseline mild neurologic disease.

However, recent work highlighted that neurological complication, including seizures, is rare after the initiation of ranolazine [210]. Since delirium was noted as a possible rare side effect [211], case reports could provide additional information to determine the optimal regimen for elderly patients and individuals with renal impairment [209]. Indeed, ranolazine inhibits the tubular secretion of creatinine but does not affect the glomerular filtration rate (https://www.ncbi.nlm.nih.gov/books/NBK507828/ accessed on 10 December 2021). However, acute renal failure has been reported in patients with severe renal impairment (creatinine clearance lower than 30 mL per min). Ranolazine should be discontinued in patients with renal failure, and it is contraindicated in patients with creatinine clearance lower than 30 mL per min (e.g., patients on dialysis) and in cirrhotic patients (https://www.ncbi.nlm.nih.gov/books/NBK507828/ accessed on 10 December 2021). Considering renal failure or hepatic impairment, two Phase I, randomized, open-label studies, have been conducted to assess the effect of ranolazine pharmacokinetics in patients with poor hepatic and poor renal function [212]. The two studies revealed that the concentration of ranolazine increases by about 50% in patients with mild, moderate, and severe renal impairment and by about 75% in patients with moderate hepatic impairment. In healthy patients, slightly elevated blood urea nitrogen (BUN) and serum creatinine levels were reported without renal toxicity and are considered reversible [213].

Ranolazine is metabolized in the liver by CYP3A4 and CYP2D6. Thus, co-administration of their inhibitors (ketoconazole, macrolides, clarithromycin, ritonavir), diltiazem, fluconazole, erythromycin, and verapamil (CYP3A4) can affect ranolazine’s clearance to increase plasma level (up to three times). Therefore, it is contraindicated unless the dose is adjusted [214], as in combination with tricyclic antidepressants and some antipsychotics [215]. Numerous immunosuppressants, like sirolimus, are also a substrate for CYP3A4. Drug interaction with ranolazine has been considered to avoid a toxic accumulation of the drugs (notably in kidney transplant patients) [216]. Hepatic impairment may also increase plasma concentrations and QT lengthening via I_Kr_ inhibition. Administration of ranolazine in patients with a family history of long QT syndrome or with known prolonged QT interval should be carefully considered. Patients initiated on ranolazine should undergo a baseline ECG with follow-up monitoring of QT interval.

A dose-related interaction between ranolazine and metformin, two drugs frequently co-administered in subjects with chronic angina and co-morbid type 2 diabetes mellitus, was also observed [217]. Ranolazine may also increase serum digoxin levels by 1.5 times, leading to reducing digoxin dosage in patients who are taking both drugs [3]. Ranolazine can also increase simvastatin Cmax approximately two-fold [218].

In contrast, trials testing ranolazine combined with amiodarone/dronedarone [47,48,49] or even ivabradine [50] showed good tolerance and safety. Similarly, twice-daily doses of ranolazine increased exercise capacity and provided additional antianginal relief to symptomatic patients with severe chronic angina under standard doses of atenolol, amlodipine, or diltiazem with no evidence for adverse consequences during 1 to 2 years of therapy [17].

## 6. Conclusions

**Figure 4:**
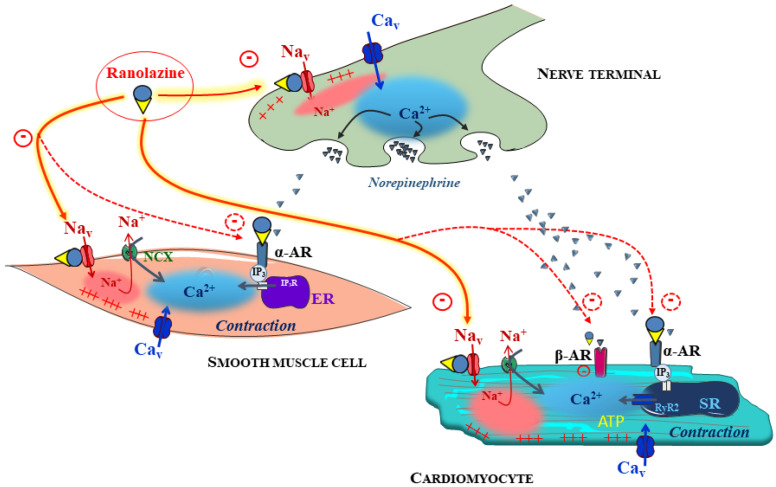
Integrated vision of ranolazine’s putative direct and indirect effects on the cardiovascular system. The therapeutic benefits of ranolazine in the treatment of ischemia and angina pectoris have been documented as primarily based on the inhibition of I_Na,late_ in heart cells. However, there is mounting evidence that ranolazine also interacts with a broad spectrum of Na_v_ channels, including cardiac and neuronal isoforms. Reports from the literature point to similar mechanisms in vascular smooth muscle cells and neurons, opening the possibility that these effects may contribute to the overall anti-ischemic effect of ranolazine. Combined α_1_-adrenergic receptor antagonization and inhibition of the Na_v_ channels of vascular smooth muscle cells may account for vascular effects of ranolazine. At the sympathetic perivascular nerve endings, ranolazine may potentially reduce electrical activity and inhibit the release of norepinephrine, in addition to the inhibition of α_1_-adrenergic receptors, which may also be relevant for the antianginal effects of the drug. The inhibition of cardiac release of norepinephrine may also have favorable effects by reducing cardiac adrenergic stimulation and improving ATP consumption. These complementary effects have to be confirmed from molecular targets to integrative models.

Ranolazine has multiple molecular targets on cardiac cells and pleiotropic biological actions with novel challenging findings that it also affects vessels, neurons (Figure 4), and other organs and parameters (e.g., gluco-metabolism, skeletal muscles). These properties may be fascinating in preventing adverse cardiovascular outcomes by acting on metabolism, glycemia, vasculature, autonomic nervous system, and Na^+^ transports. All these systems or functions are rarely altered independently of each other and form a continuum of the pathologic progression leading to fatal events. This is the case in ischemic and chronic heart diseases of different etiologies related to diabetes, obesity, or heart failure. Acting on several parameters with one compound able to work on numerous interrelated “functions” could be desirable, and the notion of a dirty drug should be re-evaluated since ranolazine seems well tolerated in patients. In addition to its antianginal clinical indications, a large amount of scientific evidence in the literature and clinical observations call for a repositioning of this drug as an antiarrhythmic, in pulmonary arterial hypertension, in myotonia, or even in diabetes, even if the link between the different molecular effects of ranolazine and the empirically observed benefits is complex and most often unclear. More precise pharmacological approaches and an integrative vision of the multiple effects of this molecule (like others) could open up new perspectives for its clinical use, particularly in the context of declining or leveling off the results of R&D programs. Thus, this strategy could take advantage of the untapped potential of older drugs that are sometimes poorly studied or on a variable too specific for a more complex problem. The combined use of ancient molecules, such as ranolazine, could help gain efficiency or target new indications while reducing the side effects. This can be achieved, for example, by varying the doses administered as for dronedarone/amiodarone or dofetilide for arrhythmia.

## Data Availability

Not applicable.

## Supplementary Materials

The following are available online at https://www.mdpi.com/article/10.3390/ph15010031/s1, Table S1: Summary of main experimental studies of ranolazine; Table S2: Summary of main clinical studies of ranolazine.
