# Supplementary material for: Ranolazine: An Old Drug with Emerging Potential; Lessons from Pre-Clinical and Clinical Investigations for Possible Repositioning
Source: Pharmaceuticals (Basel). 2021 Dec 25;15(1):31. doi: 10.3390/ph15010031 (PMC8777683; doi:10.3390/ph15010031)
Supplement: Supplementary file 1 [file pharmaceuticals-15-00031-s001.zip › pharmaceuticals-1437488-Table S1.pdf]

Table S1. Summary of main experimental studies of ranolazine.

Anti-Angina/Ischemia

Mode of action	*Mechanism*	*Targets*	*Ref*
Improvement of metabolism	- Shift of energy production from free fatty acid to glucose oxidation by inhibiting fatty acid. Increase in efficiency of ATP production	Inhibition of 3-ketoacyl coenzyme A thiolase / activation of the pyruvate deshydrogénase	[1–12]
Improvement of diastolic function	-Ionic homeostasis (Sodium, Calcium)	Block of $I_{Na,late}$	[13–15]
	-Improvement of relaxation	Direct modulation of myofilament Ca^{2+} sensitivity.	[16]

Atrial Anti-Arrhythmics

Mode of action	*Mechanism*	*Targets*	*Ref*
Decrease atrial excitability	- APD lengthening, prolongation of ERP, increase in the excitation threshold during diastole and lengthening of conduction time / Decrease of EAD / DAD.	Block of $I_{Na,peak}$ and $I_{Na,late}$	[17–24]
	-Decrease in ectopic activity in pulmonary vein sleeve / Decrease of EAD / DAD	Block of I_{Na}	[25]

Ventricular Anti-Arrhythmic

Mode of action	*Mechanism*	*Targets*	*Ref*
Decrease ventricular excitability	- APD shortening and reduction of the dispersion of ventricular repolarization and beat-to-beat variability of APD / Decrease of EAD	Block of $I_{Na,late}$	[26–34]
	-Prolongation of ERP	Block of $I_{Na,late}$	[32,35–38]
Decrease ventricular excitability	-Indirect normalization of intracellular Ca^{2+} / Decrease of DAD	Block of $I_{Na,late}$	[15,39–45]

Autonomic Modulation

Mode of action	*Mechanism*	*Targets*	*Ref*
Normalization of cardiac autonomic control	-Possible direct effect on the autonomic nervous system	Possible involvement of Na_v blocking	[46–48]
		Undetermined	[49]
	-Normalization of alpha-1 adrenergic receptor -Adrenergic receptors antagonization	Beta-1 and Beta-2 adrenergic receptors	[50,51]

Genetic Epilepsy

Mode of action	*Mechanism*	*Targets*	*Ref*
Decrease of abnormal neuronal firing	-Inhibition of persistent Na current	Block of $Na_v1.1$ (neuronal)	[52]
	-Inhibition of persistent Na current	Block of $I_{Na,late}$	[53]
Decrease seizure frequency in a mouse model of epilepsy	-Attenuation of abnormal AP firing in pyramidal neurons.		[54]

Neuropathic Pain

Mode of action	*Mechanism*	*Targets*	*Ref*
Decrease of ectopic activation	Inhibition of persistent Na current	Block of $Na_v1.7$ (neuronal)	[55–58]

Beneficial Vascular Effects

Mode of action	*Mechanism*	*Targets*	*Ref*
Improvement of tissue perfusion	Smooth muscle cells Relaxation	Na_v and anti-alpha-1 adrenergic receptor	[59–66]

Diabetes and related diseases

Mode of action	*Mechanism*	*Targets*	*Ref*
Normalization of hemoglobin A(1c)	Increase in β-cell survival and improvement of glucose homeostasis	Undetermined	[67–71]

Pulmonary art. hypertension

Mode of action	*Mechanism*	*Targets*	*Ref*
Improvement of oxygen supply	Improvement of pulmonary hemodynamics / Anti-remodeling effect (right ventricle)	Block of $I_{Na,late}$	[66,72,73]

(Para)Myotonia congenita

Mode of action	*Mechanism*	*Targets*	*Ref*
Improvement of muscle stiffness	Inhibition of the sustained and excessive firing of skeletal muscle AP	Block of $I_{Na,late}$	[74–76]

Oncology			
Mode of action	*Mechanism*	*Targets*	*Ref*
Reduction of cancer progression	Decrease in invasiveness and cell metastasis	Block of Na_v channel (mainly $Na_v1.7$, $Na_v1.6$, and $Na_v1.5$), $I_{Na,late}$	[77,78]
		Limitation of Lipid metabolism	[79]
Cardioprotective effect	Improvement of diastolic function	Decreased ROS production and oxidative stress	[80–82]

References

1. Fragasso, G.; Spoladore, R.; Cuko, A.; Palloshi, A. Modulation of Fatty Acids Oxidation in Heart Failure by Selective Pharmacological Inhibition of 3-Ketoacyl Coenzyme-A Thiolase. *Curr. Clin. Pharmacol.* **2007**, *2*, 190–196, doi:10.2174/157488407781668776.
2. Lopaschuk, G.D.; Barr, R.; Thomas, P.D.; Dyck, J.R.B. Beneficial Effects of Trimetazidine in Ex Vivo Working Ischemic Hearts Are Due to a Stimulation of Glucose Oxidation Secondary to Inhibition of Long-Chain 3-Ketoacyl Coenzyme a Thiolase. *Circ. Res.* **2003**, *93*, e33-37, doi:10.1161/01.RES.0000086964.07404.A5.
3. Kantor, P.F.; Lucien, A.; Kozak, R.; Lopaschuk, G.D. The Antianginal Drug Trimetazidine Shifts Cardiac Energy Metabolism from Fatty Acid Oxidation to Glucose Oxidation by Inhibiting Mitochondrial Long-Chain 3-Ketoacyl Coenzyme A Thiolase. *Circ. Res.* **2000**, *86*, 580–588, doi:10.1161/01.res.86.5.580.
4. Zhao, G.; Walsh, E.; Shryock, J.C.; Messina, E.; Wu, Y.; Zeng, D.; Xu, X.; Ochoa, M.; Baker, S.P.; Hintze, T.H.; et al. Antiadrenergic and Hemodynamic Effects of Ranolazine in Conscious Dogs. *J. Cardiovasc. Pharmacol.* **2011**, *57*, 639–647, doi:10.1097/FJC.0b013e31821458e8.
5. Clarke, B.; Wyatt, K.M.; McCormack, J.G. Ranolazine Increases Active Pyruvate Dehydrogenase in Perfused Normoxic Rat Hearts: Evidence for an Indirect Mechanism. *J. Mol. Cell. Cardiol.* **1996**, *28*, 341–350, doi:10.1006/jmcc.1996.0032.
6. McCormack, J.G.; Barr, R.L.; Wolff, A.A.; Lopaschuk, G.D. Ranolazine Stimulates Glucose Oxidation in Normoxic, Ischemic, and Reperfused Ischemic Rat Hearts. *Circulation* **1996**, *93*, 135–142, doi:10.1161/01.cir.93.1.135.
7. Clarke, B.; Spedding, M.; Patmore, L.; McCormack, J.G. Protective Effects of Ranolazine in Guinea-Pig Hearts during Low-Flow Ischaemia and Their Association with Increases in Active Pyruvate Dehydrogenase. *Br. J. Pharmacol.* **1993**, *109*, 748–750, doi:10.1111/j.1476-5381.1993.tb13637.x.
8. Gralinski, M.R.; Black, S.C.; Kilgore, K.S.; Chou, A.Y.; McCormack, J.G.; Lucchesi, B.R. Cardioprotective Effects of Ranolazine (RS-43285) in the Isolated Perfused Rabbit Heart. *Cardiovasc. Res.* **1994**, *28*, 1231–1237, doi:10.1093/cvr/28.8.1231.
9. Maruyama, K.; Hara, A.; Hashizume, H.; Ushikubi, F.; Abiko, Y. Ranolazine Attenuates Palmitoyl-L-Carnitine-Induced Mechanical and Metabolic Derangement in the Isolated, Perfused Rat Heart. *J. Pharm. Pharmacol.* **2000**, *52*, 709–715, doi:10.1211/0022357001774381.
10. Matsumura, H.; Hara, A.; Hashizume, H.; Maruyama, K.; Abiko, Y. Protective Effects of Ranolazine, a Novel Anti-Ischemic Drug, on the Hydrogen Peroxide-Induced Derangements in Isolated, Perfused Rat Heart: Comparison with Dichloroacetate. *Jpn. J. Pharmacol.* **1998**, *77*, 31–39, doi:10.1254/jjp.77.31.
11. Al Batran, R.; Gopal, K.; Aburasayn, H.; Eshreif, A.; Almutairi, M.; Greenwell, A.A.; Campbell, S.A.; Saleme, B.; Court, E.A.; Eaton, F.; et al. The Antianginal Ranolazine Mitigates Obesity-Induced Nonalcoholic Fatty Liver Disease and Increases Hepatic Pyruvate Dehydrogenase Activity. *JCI Insight* **2019**, *4*, doi:10.1172/jci.insight.124643.
12. McCormack, J.G.; Stanley, W.C.; Wolff, A.A. Ranolazine: A Novel Metabolic Modulator for the Treatment of Angina. *Gen. Pharmacol.* **1998**, *30*, 639–645, doi:10.1016/s0306-3623(97)00301-7.

13. Belardinelli, L.; Shryock, J.C.; Fraser, H. Inhibition of the Late Sodium Current as a Potential Cardioprotective Principle: Effects of the Late Sodium Current Inhibitor Ranolazine. *Heart Br. Card. Soc.* **2006**, *92 Suppl 4*, iv6–iv14, doi:10.1136/hrt.2005.078790.
14. Hasenfuss, G.; Maier, L.S. Mechanism of Action of the New Anti-Ischemia Drug Ranolazine. *Clin. Res. Cardiol. Off. J. Ger. Card. Soc.* **2008**, *97*, 222–226, doi:10.1007/s00392-007-0612-y.
15. Fraser, H.; Belardinelli, L.; Wang, L.; Light, P.E.; McVeigh, J.J.; Clanachan, A.S. Ranolazine Decreases Diastolic Calcium Accumulation Caused by ATX-II or Ischemia in Rat Hearts. *J. Mol. Cell. Cardiol.* **2006**, *41*, 1031–1038, doi:10.1016/j.yjmcc.2006.08.012.
16. Lovelock, J.D.; Monasky, M.M.; Jeong, E.-M.; Lardin, H.A.; Liu, H.; Patel, B.G.; Taglieri, D.M.; Gu, L.; Kumar, P.; Pokhrel, N.; et al. Ranolazine Improves Cardiac Diastolic Dysfunction through Modulation of Myofilament Calcium Sensitivity. *Circ. Res.* **2012**, *110*, 841–850, doi:10.1161/CIRCRESAHA.111.258251.
17. Sossalla, S.; Kallmeyer, B.; Wagner, S.; Mazur, M.; Maurer, U.; Toischer, K.; Schmitto, J.D.; Seipelt, R.; Schöndube, F.A.; Hasenfuss, G.; et al. Altered Na(+) Currents in Atrial Fibrillation Effects of Ranolazine on Arrhythmias and Contractility in Human Atrial Myocardium. *J. Am. Coll. Cardiol.* **2010**, *55*, 2330–2342, doi:10.1016/j.jacc.2009.12.055.
18. Burashnikov, A.; Di Diego, J.M.; Zygmunt, A.C.; Belardinelli, L.; Antzelevitch, C. Atrium-Selective Sodium Channel Block as a Strategy for Suppression of Atrial Fibrillation: Differences in Sodium Channel Inactivation between Atria and Ventricles and the Role of Ranolazine. *Circulation* **2007**, *116*, 1449–1457, doi:10.1161/CIRCULATIONAHA.107.704890.
19. Nesterenko, V.V.; Zygmunt, A.C.; Rajamani, S.; Belardinelli, L.; Antzelevitch, C. Mechanisms of Atrial-Selective Block of Na^+ Channels by Ranolazine: II. Insights from a Mathematical Model. *Am. J. Physiol. Heart Circ. Physiol.* **2011**, *301*, H1615-1624, doi:10.1152/ajpheart.00243.2011.
20. Rajamani, S.; El-Bizri, N.; Shryock, J.C.; Makielski, J.C.; Belardinelli, L. Use-Dependent Block of Cardiac Late Na(+) Current by Ranolazine. *Heart Rhythm* **2009**, *6*, 1625–1631, doi:10.1016/j.hrthm.2009.07.042.
21. Carvas, M.; Nascimento, B.C.G.; Acar, M.; Nearing, B.D.; Belardinelli, L.; Verrier, R.L. Intrapericardial Ranolazine Prolongs Atrial Refractory Period and Markedly Reduces Atrial Fibrillation Inducibility in the Intact Porcine Heart. *J. Cardiovasc. Pharmacol.* **2010**, *55*, 286–291, doi:10.1097/FJC.0b013e3181d26416.
22. Song, Y.; Shryock, J.C.; Belardinelli, L. A Slowly Inactivating Sodium Current Contributes to Spontaneous Diastolic Depolarization of Atrial Myocytes. *Am. J. Physiol. Heart Circ. Physiol.* **2009**, *297*, H1254-1262, doi:10.1152/ajpheart.00444.2009.
23. Song, Y.; Shryock, J.C.; Belardinelli, L. An Increase of Late Sodium Current Induces Delayed Afterdepolarizations and Sustained Triggered Activity in Atrial Myocytes. *Am. J. Physiol. Heart Circ. Physiol.* **2008**, *294*, H2031-2039, doi:10.1152/ajpheart.01357.2007.
24. Bhimani, A.A.; Yasuda, T.; Sadrpour, S.A.; Khrestian, C.M.; Lee, S.; Zeng, D.; Belardinelli, L.; Waldo, A.L. Ranolazine Terminates Atrial Flutter and Fibrillation in a Canine Model. *Heart Rhythm* **2014**, *11*, 1592–1599, doi:10.1016/j.hrthm.2014.05.038.
25. Sicouri, S.; Glass, A.; Belardinelli, L.; Antzelevitch, C. Antiarrhythmic Effects of Ranolazine in Canine Pulmonary Vein Sleeve Preparations. *Heart Rhythm* **2008**, *5*, 1019–1026, doi:10.1016/j.hrthm.2008.03.018.
26. Antzelevitch, C.; Belardinelli, L.; Zygmunt, A.C.; Burashnikov, A.; Di Diego, J.M.; Fish, J.M.; Cordeiro, J.M.; Thomas, G. Electrophysiological Effects of Ranolazine, a Novel Antianginal Agent with Antiarrhythmic Properties. *Circulation* **2004**, *110*, 904–910, doi:10.1161/01.CIR.0000139333.83620.5D.
27. Antzelevitch, C.; Belardinelli, L. The Role of Sodium Channel Current in Modulating Transmural Dispersion of Repolarization and Arrhythmogenesis. *J. Cardiovasc. Electrophysiol.* **2006**, *17 Suppl 1*, S79–S85, doi:10.1111/j.1540-8167.2006.00388.x.
28. Undrovinas, A.I.; Belardinelli, L.; Undrovinas, N.A.; Sabbah, H.N. Ranolazine Improves Abnormal Repolarization and Contraction in Left Ventricular Myocytes of Dogs with Heart Failure by Inhibiting Late Sodium Current. *J. Cardiovasc. Electrophysiol.* **2006**, *17 Suppl 1*, S169–S177, doi:10.1111/j.1540-8167.2006.00401.x.

29. Qi, D.; Yang, Z.; Robinson, V.M.; Li, J.; Gao, C.; Guo, D.; Kowey, P.R.; Yan, G.-X. Heterogeneous Distribution of INa-L Determines Interregional Differences in Rate Adaptation of Repolarization. *Heart Rhythm* **2015**, *12*, 1295–1303, doi:10.1016/j.hrthm.2015.02.013.
30. Zygmunt, A.C.; Eddlestone, G.T.; Thomas, G.P.; Nesterenko, V.V.; Antzelevitch, C. Larger Late Sodium Conductance in M Cells Contributes to Electrical Heterogeneity in Canine Ventricle. *Am. J. Physiol. Heart Circ. Physiol.* **2001**, *281*, H689-697, doi:10.1152/ajpheart.2001.281.2.H689.
31. Antoons, G.; Oros, A.; Beekman, J.D.M.; Engelen, M.A.; Houtman, M.J.C.; Belardinelli, L.; Stengl, M.; Vos, M.A. Late Na(+) Current Inhibition by Ranolazine Reduces Torsades de Pointes in the Chronic Atrioventricular Block Dog Model. *J. Am. Coll. Cardiol.* **2010**, *55*, 801–809, doi:10.1016/j.jacc.2009.10.033.
32. Kumar, K.; Nearing, B.D.; Bartoli, C.R.; Kwaku, K.F.; Belardinelli, L.; Verrier, R.L. Effect of Ranolazine on Ventricular Vulnerability and Defibrillation Threshold in the Intact Porcine Heart. *J. Cardiovasc. Electrophysiol.* **2008**, *19*, 1073–1079, doi:10.1111/j.1540-8167.2008.01204.x.
33. Zhao, Z.; Fefelova, N.; Shanmugam, M.; Bishara, P.; Babu, G.J.; Xie, L.-H. Angiotensin II Induces Afterdepolarizations via Reactive Oxygen Species and Calmodulin Kinase II Signaling. *J. Mol. Cell. Cardiol.* **2011**, *50*, 128–136, doi:10.1016/j.yjmcc.2010.11.001.
34. Wu, L.; Rajamani, S.; Li, H.; January, C.T.; Shryock, J.C.; Belardinelli, L. Reduction of Repolarization Reserve Unmasks the Proarrhythmic Role of Endogenous Late Na(+) Current in the Heart. *Am. J. Physiol. Heart Circ. Physiol.* **2009**, *297*, H1048-1057, doi:10.1152/ajpheart.00467.2009.
35. Nieminen, T.; Nanbu, D.Y.; Datti, I.P.; Vaz, G.R.; Tavares, C.A.M.; Pegler, J.R.M.; Nearing, B.D.; Belardinelli, L.; Verrier, R.L. Antifibrillatory Effect of Ranolazine during Severe Coronary Stenosis in the Intact Porcine Model. *Heart Rhythm* **2011**, *8*, 608–614, doi:10.1016/j.hrthm.2010.11.029.
36. Dhalla, A.K.; Wang, W.-Q.; Dow, J.; Shryock, J.C.; Belardinelli, L.; Bhandari, A.; Kloner, R.A. Ranolazine, an Antianginal Agent, Markedly Reduces Ventricular Arrhythmias Induced by Ischemia and Ischemia-Reperfusion. *Am. J. Physiol. Heart Circ. Physiol.* **2009**, *297*, H1923-1929, doi:10.1152/ajpheart.00173.2009.
37. Wei, X.; Zhu, A.; Zhang, Y.; Yao, S.; Mao, W. Pre- and Delayed Treatments With Ranolazine Ameliorate Ventricular Arrhythmias and Nav1.5 Downregulation in Ischemic/Reperfused Rat Hearts. *J. Cardiovasc. Pharmacol.* **2016**, *68*, 269–279, doi:10.1097/FJC.0000000000000412.
38. Morita, N.; Lee, J.H.; Xie, Y.; Sovari, A.; Qu, Z.; Weiss, J.N.; Karagueuzian, H.S. Suppression of Re-Entrant and Multifocal Ventricular Fibrillation by the Late Sodium Current Blocker Ranolazine. *J. Am. Coll. Cardiol.* **2011**, *57*, 366–375, doi:10.1016/j.jacc.2010.07.045.
39. Wang, W.-Q.; Robertson, C.; Dhalla, A.K.; Belardinelli, L. Antitorsadogenic Effects of ({+/-})-N-(2,6-Dimethyl-Phenyl)-(4[2-Hydroxy-3-(2-Methoxyphenoxy)Propyl]-1-Piperazine (Ranolazine) in Anesthetized Rabbits. *J. Pharmacol. Exp. Ther.* **2008**, *325*, 875–881, doi:10.1124/jpet.108.137729.
40. Sossalla, S.; Wallisch, N.; Toischer, K.; Sohns, C.; Vollmann, D.; Seegers, J.; Lüthje, L.; Maier, L.S.; Zabel, M. Effects of Ranolazine on Torsades de Pointes Tachycardias in a Healthy Isolated Rabbit Heart Model. *Cardiovasc. Ther.* **2014**, *32*, 170–177, doi:10.1111/1755-5922.12078.
41. Toischer, K.; Hartmann, N.; Wagner, S.; Fischer, T.H.; Herting, J.; Danner, B.C.; Sag, C.M.; Hund, T.J.; Mohler, P.J.; Belardinelli, L.; et al. Role of Late Sodium Current as a Potential Arrhythmogenic Mechanism in the Progression of Pressure-Induced Heart Disease. *J. Mol. Cell. Cardiol.* **2013**, *61*, 111–122, doi:10.1016/j.yjmcc.2013.03.021.
42. Viatchenko-Karpinski, S.; Kornyeyev, D.; El-Bizri, N.; Budas, G.; Fan, P.; Jiang, Z.; Yang, J.; Anderson, M.E.; Shryock, J.C.; Chang, C.-P.; et al. Intracellular Na+ Overload Causes Oxidation of CaMKII and Leads to Ca2+ Mishandling in Isolated Ventricular Myocytes. *J. Mol. Cell. Cardiol.* **2014**, *76*, 247–256, doi:10.1016/j.yjmcc.2014.09.009.
43. Sag, C.M.; Mallwitz, A.; Wagner, S.; Hartmann, N.; Schotola, H.; Fischer, T.H.; Ungeheuer, N.; Herting, J.; Shah, A.M.; Maier, L.S.; et al. Enhanced Late INa Induces Proarrhythmogenic SR Ca Leak in a CaMKII-Dependent Manner. *J. Mol. Cell. Cardiol.* **2014**, *76*, 94–105, doi:10.1016/j.yjmcc.2014.08.016.

44. Undrovinas, N.A.; Maltsev, V.A.; Belardinelli, L.; Sabbah, H.N.; Undrovinas, A. Late Sodium Current Contributes to Diastolic Cell Ca2+ Accumulation in Chronic Heart Failure. *J. Physiol. Sci. JPS* **2010**, *60*, 245–257, doi:10.1007/s12576-010-0092-0.
45. Wasserstrom, J.A.; Sharma, R.; O'Toole, M.J.; Zheng, J.; Kelly, J.E.; Shryock, J.; Belardinelli, L.; Aistrup, G.L. Ranolazine Antagonizes the Effects of Increased Late Sodium Current on Intracellular Calcium Cycling in Rat Isolated Intact Heart. *J. Pharmacol. Exp. Ther.* **2009**, *331*, 382–391, doi:10.1124/jpet.109.156471.
46. Murray, G.L. Ranolazine Is an Effective and Safe Treatment of Adults with Symptomatic Premature Ventricular Contractions Due to Triggered Ectopy. *Int. J. Angiol. Off. Publ. Int. Coll. Angiol. Inc* **2016**, *25*, 247–251, doi:10.1055/s-0036-1584880.
47. Murray, G.L.; Colombo, J. Ranolazine Preserves and Improves Left Ventricular Ejection Fraction and Autonomic Measures When Added to Guideline-Driven Therapy in Chronic Heart Failure. *Heart Int.* **2014**, *9*, 66–73, doi:10.5301/heartint.5000219.
48. Feng, G.; Yang, Y.; Chen, J.; Wu, Z.; Zheng, Y.; Li, W.; Dai, W.; Guan, P.; Zhong, C. Ranolazine Attenuated Heightened Plasma Norepinephrine and B-Type Natriuretic Peptide-45 in Improving Cardiac Function in Rats with Chronic Ischemic Heart Failure. *Am. J. Transl. Res.* **2016**, *8*, 1295–1301.
49. Allely, M.C.; Brown, C.M.; Kenny, B.A.; Kilpatrick, A.T.; Martin, A.; Spedding, M. Modulation of Alpha 1-Adrenoceptors in Rat Left Ventricle by Ischaemia and Acyl Carnitines: Protection by Ranolazine. *J. Cardiovasc. Pharmacol.* **1993**, *21*, 869–873, doi:10.1097/00005344-199306000-00004.
50. Létienne, R.; Vié, B.; Puech, A.; Vieu, S.; Le Grand, B.; John, G.W. Evidence That Ranolazine Behaves as a Weak Beta1- and Beta2-Adrenoceptor Antagonist in the Rat [Correction of Cat] Cardiovascular System. *Naunyn. Schmiedebergs Arch. Pharmacol.* **2001**, *363*, 464–471, doi:10.1007/s002100000378.
51. Flenner, F.; Friedrich, F.W.; Ungeheuer, N.; Christ, T.; Geertz, B.; Reischmann, S.; Wagner, S.; Stathopoulou, K.; Söhren, K.-D.; Weinberger, F.; et al. Ranolazine Antagonizes Catecholamine-Induced Dysfunction in Isolated Cardiomyocytes, but Lacks Long-Term Therapeutic Effects in Vivo in a Mouse Model of Hypertrophic Cardiomyopathy. *Cardiovasc. Res.* **2016**, *109*, 90–102, doi:10.1093/cvr/cvv247.
52. Kahlig, K.M.; Lepist, I.; Leung, K.; Rajamani, S.; George, A.L. Ranolazine Selectively Blocks Persistent Current Evoked by Epilepsy-Associated Nav1.1 Mutations. *Br. J. Pharmacol.* **2010**, *161*, 1414–1426, doi:10.1111/j.1476-5381.2010.00976.x.
53. Peters, C.H.; Sokolov, S.; Rajamani, S.; Ruben, P.C. Effects of the Antianginal Drug, Ranolazine, on the Brain Sodium Channel Na(V)1.2 and Its Modulation by Extracellular Protons. *Br. J. Pharmacol.* **2013**, *169*, 704–716, doi:10.1111/bph.12150.
54. Anderson, L.L.; Thompson, C.H.; Hawkins, N.A.; Nath, R.D.; Petersohn, A.A.; Rajamani, S.; Bush, W.S.; Frankel, W.N.; Vanoye, C.G.; Kearney, J.A.; et al. Antiepileptic Activity of Preferential Inhibitors of Persistent Sodium Current. *Epilepsia* **2014**, *55*, 1274–1283, doi:10.1111/epi.12657.
55. Estacion, M.; Waxman, S.G.; Dib-Hajj, S.D. Effects of Ranolazine on Wild-Type and Mutant HNav1.7 Channels and on DRG Neuron Excitability. *Mol. Pain* **2010**, *6*, 35, doi:10.1186/1744-8069-6-35.
56. Wang, G.K.; Calderon, J.; Wang, S.-Y. State- and Use-Dependent Block of Muscle Nav1.4 and Neuronal Nav1.7 Voltage-Gated Na+ Channel Isoforms by Ranolazine. *Mol. Pharmacol.* **2008**, *73*, 940–948, doi:10.1124/mol.107.041541.
57. Theile, J.W.; Cummins, T.R. Recent Developments Regarding Voltage-Gated Sodium Channel Blockers for the Treatment of Inherited and Acquired Neuropathic Pain Syndromes. *Front. Pharmacol.* **2011**, *2*, doi:10.3389/fphar.2011.00054.
58. Gould, H.J.; Garrett, C.; Donahue, R.R.; Paul, D.; Diamond, I.; Taylor, B.K. Ranolazine Attenuates Behavioral Signs of Neuropathic Pain. *Behav. Pharmacol.* **2009**, *20*, 755–758, doi:10.1097/FBP.0b013e3283323c90.

59. Paredes-Carbajal, M.C.; Monsalvo, I.; Hernández-Díaz, C.; Regla, I.; Demare, P.; Mascher, D. Effects of Ranolazine on Vasomotor Responses of Rat Aortic Rings. *Arch. Med. Res.* **2013**, *44*, 8–12, doi:10.1016/j.arcmed.2012.11.002.
60. A, V.; C, F.; N, P.; L, K.; A, L.; C, R.; F, A.; S, R. Antagonism of Nav Channels and A1-Adrenergic Receptors Contributes to Vascular Smooth Muscle Effects of Ranolazine. *Sci. Rep.* **2015**, *5*, doi:10.1038/srep17969.
61. Khazraei, H.; Shafa, M.; Mirkhani, H. Effect of Ranolazine on Cardiac Microcirculation in Normal and Diabetic Rats. *Acta Physiol. Hung.* **2014**, *101*, 301–308, doi:10.1556/APhysiol.101.2014.3.5.
62. Malavaki, C.; Hatziefthimiou, A.; Daskalopoulou, S.S.; Stefanidis, I.; Karatzaferi, C.; Aidonidis, I. Ranolazine Enhances Nicardipine-Induced Relaxation of Alpha1-Adrenoceptor-Mediated Contraction on Isolated Rabbit Aorta. *Acta Cardiol.* **2015**, *70*, 157–162, doi:10.1080/ac.70.2.3073506.
63. Ahmed, B.; Mondragon, J.; Sheldon, M.; Clegg, S. Impact of Ranolazine on Coronary Microvascular Dysfunction (MICRO) Study. *Cardiovasc. Revascularization Med. Mol. Interv.* **2017**, *18*, 431–435, doi:10.1016/j.carrev.2017.04.012.
64. Rambarat, C.A.; Elgendy, I.Y.; Handberg, E.M.; Bairey Merz, C.N.; Wei, J.; Minissian, M.B.; Nelson, M.D.; Thomson, L.E.J.; Berman, D.S.; Shaw, L.J.; et al. Late Sodium Channel Blockade Improves Angina and Myocardial Perfusion in Patients with Severe Coronary Microvascular Dysfunction: Women's Ischemia Syndrome Evaluation-Coronary Vascular Dysfunction Ancillary Study. *Int. J. Cardiol.* **2019**, *276*, 8–13, doi:10.1016/j.ijcard.2018.09.081.
65. Zhu, H.; Xu, X.; Fang, X.; Zheng, J.; Zhao, Q.; Chen, T.; Huang, J. Effects of the Antianginal Drugs Ranolazine, Nicorandil, and Ivabradine on Coronary Microvascular Function in Patients With Nonobstructive Coronary Artery Disease: A Meta-Analysis of Randomized Controlled Trials. *Clin. Ther.* **2019**, *41*, 2137-2152.e12, doi:10.1016/j.clinthera.2019.08.008.
66. Rocchetti, M.; Sala, L.; Rizzetto, R.; Staszewsky, L.I.; Alemanni, M.; Zambelli, V.; Russo, I.; Barile, L.; Cornaghi, L.; Altomare, C.; et al. Ranolazine Prevents INaL Enhancement and Blunts Myocardial Remodelling in a Model of Pulmonary Hypertension. *Cardiovasc. Res.* **2014**, *104*, 37–48, doi:10.1093/cvr/cvu188.
67. Ning, Y.; Zhen, W.; Fu, Z.; Jiang, J.; Liu, D.; Belardinelli, L.; Dhalla, A.K. Ranolazine Increases β-Cell Survival and Improves Glucose Homeostasis in Low-Dose Streptozotocin-Induced Diabetes in Mice. *J. Pharmacol. Exp. Ther.* **2011**, *337*, 50–58, doi:10.1124/jpet.110.176396.
68. Cassano, V.; Leo, A.; Tallarico, M.; Nesci, V.; Cimellaro, A.; Fiorentino, T.V.; Citraro, R.; Hribal, M.L.; De Sarro, G.; Perticone, F.; et al. Metabolic and Cognitive Effects of Ranolazine in Type 2 Diabetes Mellitus: Data from an in Vivo Model. *Nutrients* **2020**, *12*, doi:10.3390/nu12020382.
69. Chen, X.; Ren, L.; Liu, X.; Sun, X.; Dong, C.; Jiang, Y.; Qin, Y.; Qu, H.; Jiao, J.; Wang, S.; et al. Ranolazine Protects against Diabetic Cardiomyopathy by Activating the NOTCH1/NRG1 Pathway. *Life Sci.* **2020**, *261*, 118306, doi:10.1016/j.lfs.2020.118306.
70. Mourouzis, I.; Mantzouratou, P.; Galanopoulos, G.; Kostakou, E.; Dhalla, A.K.; Belardinelli, L.; Pantos, C. The Beneficial Effects of Ranolazine on Cardiac Function after Myocardial Infarction Are Greater in Diabetic than in Nondiabetic Rats. *J. Cardiovasc. Pharmacol. Ther.* **2014**, *19*, 457–469, doi:10.1177/1074248414524481.
71. Khazraei, H.; Akmali, M.; Mirkhani, H. Mechanical Effects of Ranolazine on Normal and Diabetic-Isolated Rat Heart. *Res. Pharm. Sci.* **2021**, *16*, 269–277, doi:10.4103/1735-5362.314825.
72. Lee, J.C.; Kim, K.C.; Choe, S.Y.; Hong, Y.M. Reduced Immunoreactivities of B-Type Natriuretic Peptide in Pulmonary Arterial Hypertension Rats after Ranolazine Treatment. *Anat. Cell Biol.* **2016**, *49*, 7–14, doi:10.5115/acb.2016.49.1.7.
73. Liles, J.T.; Hoyer, K.; Oliver, J.; Chi, L.; Dhalla, A.K.; Belardinelli, L. Ranolazine Reduces Remodeling of the Right Ventricle and Provoked Arrhythmias in Rats with Pulmonary Hypertension. *J. Pharmacol. Exp. Ther.* **2015**, *353*, 480–489, doi:10.1124/jpet.114.221861.
74. Hawash, A.A.; Voss, A.A.; Rich, M.M. Inhibiting Persistent Inward Sodium Currents Prevents Myotonia. *Ann. Neurol.* **2017**, *82*, 385–395, doi:10.1002/ana.25017.

75. El-Bizri, N.; Kahlig, K.M.; Shyrock, J.C.; George, A.L.; Belardinelli, L.; Rajamani, S. Ranolazine Block of Human Na v 1.4 Sodium Channels and Paramyotonia Congenita Mutants. *Channels Austin Tex* **2011**, *5*, 161–172, doi:10.4161/chan.5.2.14851.
76. Novak, K.R.; Norman, J.; Mitchell, J.R.; Pinter, M.J.; Rich, M.M. Sodium Channel Slow Inactivation as a Therapeutic Target for Myotonia Congenita. *Ann. Neurol.* **2015**, *77*, 320–332, doi:10.1002/ana.24331.
77. Driffort, V.; Gillet, L.; Bon, E.; Marionneau-Lambot, S.; Oullier, T.; Joulin, V.; Collin, C.; Pagès, J.-C.; Jourdan, M.-L.; Chevalier, S.; et al. Ranolazine Inhibits NaV1.5-Mediated Breast Cancer Cell Invasiveness and Lung Colonization. *Mol. Cancer* **2014**, *13*, 264, doi:10.1186/1476-4598-13-264.
78. Bugan, I.; Kucuk, S.; Karagoz, Z.; Fraser, S.P.; Kaya, H.; Dodson, A.; Foster, C.S.; Altun, S.; Djamgoz, M.B.A. Anti-Metastatic Effect of Ranolazine in an in Vivo Rat Model of Prostate Cancer, and Expression of Voltage-Gated Sodium Channel Protein in Human Prostate. *Prostate Cancer Prostatic Dis.* **2019**, *22*, 569–579, doi:10.1038/s41391-019-0128-3.
79. Guth, B.D.; Engwall, M.; Eldridge, S.; Foley, C.M.; Guo, L.; Gintant, G.; Koerner, J.; Parish, S.T.; Pierson, J.B.; Ribeiro, A.J.S.; et al. Considerations for an In Vitro, Cell-Based Testing Platform for Detection of Adverse Drug-Induced Inotropic Effects in Early Drug Development. Part 1: General Considerations for Development of Novel Testing Platforms. *Front. Pharmacol.* **2019**, *10*, 884, doi:10.3389/fphar.2019.00884.
80. Riccio, G.; Antonucci, S.; Coppola, C.; D'Avino, C.; Piscopo, G.; Fiore, D.; Maurea, C.; Russo, M.; Rea, D.; Arra, C.; et al. Ranolazine Attenuates Trastuzumab-Induced Heart Dysfunction by Modulating ROS Production. *Front. Physiol.* **2018**, *9*, 38, doi:10.3389/fphys.2018.00038.
81. Tocchetti, C.G.; Carpi, A.; Coppola, C.; Quintavalle, C.; Rea, D.; Campesan, M.; Arcari, A.; Piscopo, G.; Cipresso, C.; Monti, M.G.; et al. Ranolazine Protects from Doxorubicin-Induced Oxidative Stress and Cardiac Dysfunction. *Eur. J. Heart Fail.* **2014**, *16*, 358–366, doi:10.1002/ejhf.50.
82. Cappetta, D.; Esposito, G.; Coppini, R.; Piegari, E.; Russo, R.; Ciuffreda, L.P.; Rivellino, A.; Santini, L.; Rafaniello, C.; Scavone, C.; et al. Effects of Ranolazine in a Model of Doxorubicin-Induced Left Ventricle Diastolic Dysfunction. *Br. J. Pharmacol.* **2017**, *174*, 3696–3712, doi:10.1111/bph.13791.
