# Supplementary material for: Ranolazine: An Old Drug with Emerging Potential; Lessons from Pre-Clinical and Clinical Investigations for Possible Repositioning
Source: Pharmaceuticals (Basel). 2021 Dec 25;15(1):31. doi: 10.3390/ph15010031 (PMC8777683; doi:10.3390/ph15010031)
Supplement: Supplementary file 1 [file pharmaceuticals-15-00031-s001.zip › pharmaceuticals-1437488-Table S2.pdf]

Table S2: Summary of main clinical studies of ranolazine.

Targets/Strategies	Study design	Results	*Ref*
To determine whether ranolazine improves total exercise time of patients with symptoms of chronic angina, and angina and ischemia at low workloads despite treatment with standard doses of atenolol, amlodipine, or diltiazem and to determine times to angina onset and electrocardiographic evidence of myocardial ischemia, effect on angina attacks and nitroglycerin use, and effect on long-term survival in an open-label observational study extension.)	A randomized, 3-group parallel, double-blind, placebo-controlled trial of 823 eligible adults with symptomatic chronic angina who were randomly assigned to receive a placebo or 1 of 2 doses of ranolazine. Combination Assessment of Ranolazine In Stable Angina (CARISA	Twice-daily doses of ranolazine increased exercise capacity and provided additional anti-anginal relief to symptomatic patients with severe chronic angina taking standard doses of atenolol, amlodipine, or diltiazem, without evident adverse, long-term survival consequences over 1 to 2 years of therapy.	[1]
Monotherapy Assessment of Ranolazine In Stable Angina (MARISA) trial	Patients (n = 191) with angina-limited exercise discontinued anti-anginal medications randomized into a double-blind four-period crossover study of sustained-release ranolazine 500, 1,000, or 1,500 mg, or placebo, each administered twice daily for one week.	Monotherapy was well tolerated and increased exercise performance throughout its dosing interval at all doses studied without clinically meaningful hemodynamic effects. Ranolazine reduced HbA1c vs. placebo.	[2]
Safety and tolerability data from 746 chronic angina patients treated in the ROLE (Ranolazine Open-Label Experience) program	Patients with severe functional impairment from angina who completed 1 of 2 randomized treadmill trials entered the ROLE program. Ranolazine was titrated to optimal dosages between 500 and 1,000 mg twice daily.	Long-term therapy with ranolazine seems well tolerated in high-risk coronary heart disease patients.	[3]
Symptomatic premature ventricular contractions (PVCs) due to triggered ectopy: Safety and tolerability of Ranolazine	Fifty-nine patients with symptomatic PVCs were identified from full-disclosure Holters. Doses of 500 and 1,000 mg off label RANOLAZINE (RAN), daily, were given to 34 and 66% patients, respectively, and repeat Holters were performed prospectively during a mean follow-up of 3.1 months.	Dose-dependant PVC reduction. Off-label RAN offers an effective and safe pharmacologic treatment for symptomatic triggered PVCs	[4]
Anti-arrhythmic actions	Metabolic Efficiency With Ranolazine for Less Ischemia in Non-ST-Elevation Acute Coronary Syndrome (MERLIN)-Thrombolysis in Myocardial Infarction (TIMI) 36 (MERLIN-TIMI 36) trial randomized 6560 patients hospitalized with a non-ST-elevation acute coronary syndrome to ranolazine or placebo in addition to standard therapy.	Anti-arrhythmic effects (ventricular and atrial events), reduced angina with favorable safety, and improved exercise performance.	[5–7]
Atrial fibrillation (AF)	Patients with recurrent AF within hours to a few days of restoring sinus rhythm despite AF ablation and /or failing one or more anti-arrhythmic agents were started on ranolazine (500-1000 mg/twice/day) after stopping all other anti-arrhythmic therapy.	Ranolazine helped maintain sinus rhythm in the majority of patients in which more established measures had failed	[8–10]

Ranolazine versus amiodarone for the prevention of AF after coronary artery bypass grafting (CABG)	A retrospective cohort study of patients undergoing CABG at Aspirus Hospital from June 2008 to April 2010. The patients received either amiodarone (400 mg preoperatively followed by 200 mg twice daily for 10 to 14 days) or ranolazine (1,500 mg preoperatively followed by 1,000 mg twice daily for 10 to 14 days).	Ranolazine was independently associated with a significant reduction of AF compared to amiodarone after CABG, with no difference in the incidence of adverse events.	[11–13]
Incidence of Postoperative atrial fibrillation (POAF) following coronary artery bypass grafting (CABG), valve or combination surgeries when perioperative ranolazine (1,000 mg preoperatively, then 1,000 mg twice daily for seven days or until discharge) was or was not added to standard therapy.	Two hundred five patients were evaluated for POAF after CABG, valve, or combination surgeries.	Adding ranolazine to standard therapy was independently associated with a significant decrease in POAF development after CABG, valve, or combination surgeries.	[13]
Ranolazine administration decreases the likelihood of Ventricular tachycardia (VT), ventricular fibrillation (VF), or death in patients with an implantable cardioverter-defibrillators (ICDs).	A double-blind, placebo-controlled clinical trial in high-risk ICD patients with ischemic or nonischemic cardiomyopathy was randomized to 1,000 mg ranolazine twice a day or placebo.	Ranolazine administration was associated with a significant reduction in recurrent VT or VF requiring ICD therapy without evidence for increased mortality. (Ranolazine Implantable Cardioverter-Defibrillator Trial [RAID]; NCT01215253).	[14]
Ranolazine reduces the frequency of angina and the use of sublingual nitroglycerin (SL NTG) in stable angina patients with type 2 diabetes (T2DM).	TERISA was a multinational, randomized, double-blind trial of ranolazine vs. placebo in patients with T2DM and stable angina. Anginal episodes and SL NTG use were recorded daily in an electronic diary. Health status was evaluated at baseline and 8weeks post-randomization using the Seattle Angina Questionnaire (SAQ).	Ranolazine is particularly beneficial in patients with stable angina who have suboptimally controlled T2DM.	[15]
Anti-hyperglycemic effect of ranolazine in type 2 diabetes mellitus (T2DM).	EMBASE search was conducted between January 1966 through December 2015 using the search terms ranolazine, diabetes, and hemoglobin A1C(A1C).	For patients with T2DM and chronic stable angina, ranolazine may be of use given its utility in cardiovascular disease and benefit in A1C lowering.	[16,17]
Ranolazine is an adjunctive therapy in patients with chronic stable angina whose symptoms are inadequately controlled by conventional treatment.	Monotherapy Assessment of Ranolazine In Stable Angina (MARISA) trial, CARISA study (Combination Assessment of Ranolazine In Stable Angina), Efficacy of Ranolazine In Chronic Angina (ERICA) trial, and The MERLIN-TIMI 36 trial	Ranolazine is a non-hemodynamic anti-anginal agent effective as adjunctive therapy in patients with chronic stable angina whose symptoms are not controlled by conventional treatment. Ranolazine improves exercise performance, decreases both angina and sublingual nitrates, compared to placebo, and is well tolerated, with a neutral effect on hemodynamics.	[18]
Antihyperglycemic and metabolic effects of ranolazine.	MEDLINE was searched from 2000 to October 1, 2016, using the terms ranolazine, anti-hyperglycemic, diabetes, cardiology, and anti-anginal.	Ranolazine has been shown to have positive anti-hyperglycemic and metabolic effects in patients with uncontrolled HbA1c.	[19]
Ranolazine improves angina and coronary microvascular dysfunction	Monotherapy assessment of Ranolazine in Coronary Microvascular Dysfunction or Angina	Ranolazine improves organ perfusion, measures of angina severity, and exercise tolerance.	[20–22]

Ranolazine Improves Right Ventricular Function in Patients With Precapillary Pulmonary Hypertension	A longitudinal, randomized, double-blinded, placebo-controlled, multicenter study of ranolazine treatment in patients with pulmonary hypertension and right ventricular dysfunction PH and RV dysfunction.	Ranolazine improves cardiac remodeling (right ventricle) and susceptibility to ventricular arrhythmia in patients with precapillary pulmonary hypertension	[23–26]
Ranolazine for the treatment of myotonia or paramytotonia congenita	Open-label, pilot study to evaluate Ranolazine efficacy and tolerability in myotonia or paramytotonia congenita	Ranolazine is well tolerated in patients with myotonia or paramytotonia congenita and improves clinical signs in patients.	[27,28]
Ranolazine versus Common Cardiovascular Drugs in Patients with Early Diastolic Dysfunction Induced by Anthracyclines or Nonanthracycline	A Phase 2b Minitrial INTERACT (ranolazINe to Treat EaRly cArdiotoxiCity induced by antiTumor drugs	Ranolazine can potentially relieve early diastolic dysfunction induced by anthracyclines or non-anthracycline chemotherapeutics with a similar safety profile than in the general population.	[29]

References

1. Chaitman, B.R.; Pepine, C.J.; Parker, J.O.; Skopal, J.; Chumakova, G.; Kuch, J.; Wang, W.; Skettino, S.L.; Wolff, A.A.; Combination Assessment of Ranolazine In Stable Angina (CARISA) Investigators Effects of Ranolazine with Atenolol, Amlodipine, or Diltiazem on Exercise Tolerance and Angina Frequency in Patients with Severe Chronic Angina: A Randomized Controlled Trial. *JAMA* **2004**, *291*, 309–316, doi:10.1001/jama.291.3.309.
2. Chaitman, B.R.; Skettino, S.L.; Parker, J.O.; Hanley, P.; Meluzin, J.; Kuch, J.; Pepine, C.J.; Wang, W.; Nelson, J.J.; Hebert, D.A.; et al. Anti-Ischemic Effects and Long-Term Survival during Ranolazine Monotherapy in Patients with Chronic Severe Angina. *J. Am. Coll. Cardiol.* **2004**, *43*, 1375–1382, doi:10.1016/j.jacc.2003.11.045.
3. Koren, M.J.; Crager, M.R.; Sweeney, M. Long-Term Safety of a Novel Antianginal Agent in Patients with Severe Chronic Stable Angina: The Ranolazine Open-Label Experience (ROLE). *J. Am. Coll. Cardiol.* **2007**, *49*, 1027–1034, doi:10.1016/j.jacc.2006.10.067.
4. Murray, G.L. Ranolazine Is an Effective and Safe Treatment of Adults with Symptomatic Premature Ventricular Contractions Due to Triggered Ectopy. *Int. J. Angiol. Off. Publ. Int. Coll. Angiol. Inc* **2016**, *25*, 247–251, doi:10.1055/s-0036-1584880.
5. Wilson, S.R.; Scirica, B.M.; Braunwald, E.; Murphy, S.A.; Karwatowska-Prokopczuk, E.; Buros, J.L.; Chaitman, B.R.; Morrow, D.A. Efficacy of Ranolazine in Patients with Chronic Angina Observations from the Randomized, Double-Blind, Placebo-Controlled MERLIN-TIMI (Metabolic Efficiency With Ranolazine for Less Ischemia in Non-ST-Segment Elevation Acute Coronary Syndromes) 36 Trial. *J. Am. Coll. Cardiol.* **2009**, *53*, 1510–1516, doi:10.1016/j.jacc.2009.01.037.
6. Scirica, B.M.; Morrow, D.A.; Hod, H.; Murphy, S.A.; Belardinelli, L.; Hedgepeth, C.M.; Molhoek, P.; Verheugt, F.W.A.; Gersh, B.J.; McCabe, C.H.; et al. Effect of Ranolazine, an Antianginal Agent with Novel Electrophysiological Properties, on the Incidence of Arrhythmias in Patients with Non ST-Segment Elevation Acute Coronary Syndrome: Results from the Metabolic Efficiency With Ranolazine for Less Ischemia in Non ST-Elevation Acute Coronary Syndrome Thrombolysis in Myocardial Infarction 36 (MERLIN-TIMI 36) Randomized Controlled Trial. *Circulation* **2007**, *116*, 1647–1652, doi:10.1161/CIRCULATIONAHA.107.724880.
7. Scirica, B.M.; Belardinelli, L.; Chaitman, B.R.; Waks, J.W.; Volo, S.; Karwatowska-Prokopczuk, E.; Murphy, S.A.; Cheng, M.L.; Braunwald, E.; Morrow, D.A. Effect of Ranolazine on Atrial Fibrillation in Patients with Non-ST Elevation Acute Coronary Syndromes: Observations from the MERLIN-TIMI 36 Trial. *Eur. Eur. Pacing Arrhythm. Card. Electrophysiol. J. Work. Groups Card. Pacing Arrhythm. Card. Cell. Electrophysiol. Eur. Soc. Cardiol.* **2015**, *17*, 32–37, doi:10.1093/europace/euu217.
8. Murdock, D.K.; Overton, N.; Kersten, M.; Kaliebe, J.; Devecchi, F. The Effect of Ranolazine on Maintaining Sinus Rhythm in Patients with Resistant Atrial Fibrillation. *Indian Pacing Electrophysiol. J.* **2008**, *8*, 175–181.

9. Reiffel, J.A.; Camm, A.J.; Belardinelli, L.; Zeng, D.; Karwatowska-Prokopczuk, E.; Olmsted, A.; Zareba, W.; Rosero, S.; Kowey, P.; HARMONY Investigators The HARMONY Trial: Combined Ranolazine and Dronedarone in the Management of Paroxysmal Atrial Fibrillation: Mechanistic and Therapeutic Synergism. *Circ. Arrhythm. Electrophysiol.* **2015**, *8*, 1048–1056, doi:10.1161/CIRCEP.115.002856.
10. Murdock, D.K.; Kersten, M.; Kaliebe, J.; Larrain, G. The Use of Oral Ranolazine to Convert New or Paroxysmal Atrial Fibrillation: A Review of Experience with Implications for Possible "Pill in the Pocket" Approach to Atrial Fibrillation. *Indian Pacing Electrophysiol. J.* **2009**, *9*, 260–267.
11. Miles, R.H.; Passman, R.; Murdock, D.K. Comparison of Effectiveness and Safety of Ranolazine versus Amiodarone for Preventing Atrial Fibrillation after Coronary Artery Bypass Grafting. *Am. J. Cardiol.* **2011**, *108*, 673–676, doi:10.1016/j.amjcard.2011.04.017.
12. Tsanaxidis, N.; Aidonidis, I.; Hatziefthimiou, A.; Daskalopoulou, S.S.; Giamouzis, G.; Triposkiadis, F.; Skoularigis, I. Ranolazine Added to Amiodarone Facilitates Earlier Conversion of Atrial Fibrillation Compared to Amiodarone-Only Therapy. *Pacing Clin. Electrophysiol. PACE* **2017**, *40*, 372–378, doi:10.1111/pace.13048.
13. Hammond, D.A.; Smotherman, C.; Jankowski, C.A.; Tan, S.; Osian, O.; Kraemer, D.; DeLosSantos, M. Short-Course of Ranolazine Prevents Postoperative Atrial Fibrillation Following Coronary Artery Bypass Grafting and Valve Surgeries. *Clin. Res. Cardiol. Off. J. Ger. Card. Soc.* **2015**, *104*, 410–417, doi:10.1007/s00392-014-0796-x.
14. Zareba, W.; Daubert, J.P.; Beck, C.A.; Huang, D.T.; Alexis, J.D.; Brown, M.W.; Pyykkonen, K.; McNitt, S.; Oakes, D.; Feng, C.; et al. Ranolazine in High-Risk Patients With Implanted Cardioverter-Defibrillators: The RAID Trial. *J. Am. Coll. Cardiol.* **2018**, *72*, 636–645, doi:10.1016/j.jacc.2018.04.086.
15. Arnold, S.V.; McGuire, D.K.; Spertus, J.A.; Li, Y.; Yue, P.; Ben-Yehuda, O.; Belardinelli, L.; Jones, P.G.; Olmsted, A.; Chaitman, B.R.; et al. Effectiveness of Ranolazine in Patients with Type 2 Diabetes Mellitus and Chronic Stable Angina According to Baseline Hemoglobin A1c. *Am. Heart J.* **2014**, *168*, 457-465.e2, doi:10.1016/j.ahj.2014.06.020.
16. Greiner, L.; Hurren, K.; Brenner, M. Ranolazine and Its Effects on Hemoglobin A1C. *Ann. Pharmacother.* **2016**, *50*, 410–415, doi:10.1177/1060028016631757.
17. Teoh, I.H.; Banerjee, M. Effect of Ranolazine on Glycaemia in Adults with and without Diabetes: A Meta-Analysis of Randomised Controlled Trials. *Open Heart* **2018**, *5*, e000706, doi:10.1136/openhrt-2017-000706.
18. Rosano, G.M.C.; Vitale, C.; Volterrani, M. Pharmacological Management of Chronic Stable Angina: Focus on Ranolazine. *Cardiovasc. Drugs Ther.* **2016**, *30*, 393–398, doi:10.1007/s10557-016-6674-1.
19. Gilbert, B.W.; Sherard, M.; Little, L.; Branstetter, J.; Meister, A.; Huffman, J. Antihyperglycemic and Metabolic Effects of Ranolazine in Patients With Diabetes Mellitus. *Am. J. Cardiol.* **2018**, *121*, 509–512, doi:10.1016/j.amjcard.2017.11.021.
20. Ahmed, B.; Mondragon, J.; Sheldon, M.; Clegg, S. Impact of Ranolazine on Coronary Microvascular Dysfunction (MICRO) Study. *Cardiovasc. Revascularization Med. Mol. Interv.* **2017**, *18*, 431–435, doi:10.1016/j.carrev.2017.04.012.
21. Rambarat, C.A.; Elgendy, I.Y.; Handberg, E.M.; Bairey Merz, C.N.; Wei, J.; Minissian, M.B.; Nelson, M.D.; Thomson, L.E.J.; Berman, D.S.; Shaw, L.J.; et al. Late Sodium Channel Blockade Improves Angina and Myocardial Perfusion in Patients with Severe Coronary Microvascular Dysfunction: Women's Ischemia Syndrome Evaluation-Coronary Vascular Dysfunction Ancillary Study. *Int. J. Cardiol.* **2019**, *276*, 8–13, doi:10.1016/j.ijcard.2018.09.081.
22. Zhu, H.; Xu, X.; Fang, X.; Zheng, J.; Zhao, Q.; Chen, T.; Huang, J. Effects of the Antianginal Drugs Ranolazine, Nicorandil, and Ivabradine on Coronary Microvascular Function in Patients With Nonobstructive Coronary Artery Disease: A Meta-Analysis of Randomized Controlled Trials. *Clin. Ther.* **2019**, *41*, 2137-2152.e12, doi:10.1016/j.clinthera.2019.08.008.
23. Khan, S.S.; Cuttica, M.J.; Beussink-Nelson, L.; Kozyleva, A.; Sanchez, C.; Mkrdichian, H.; Selvaraj, S.; Dematte, J.E.; Lee, D.C.; Shah, S.J. Effects of Ranolazine on Exercise Capacity, Right Ventricular Indices, and Hemodynamic Characteristics in Pulmonary Arterial Hypertension: A Pilot Study. *Pulm. Circ.* **2015**, *5*, 547–556, doi:10.1086/682427.
24. Gomberg-Maitland, M.; Schilz, R.; Mediratta, A.; Addetia, K.; Coslet, S.; Thomeas, V.; Gillies, H.; Oudiz, R.J. Phase I Safety Study of Ranolazine in Pulmonary Arterial Hypertension. *Pulm. Circ.* **2015**, *5*, 691–700, doi:10.1086/683813.

25. Han, Y.; Forfia, P.R.; Vaidya, A.; Mazurek, J.A.; Park, M.H.; Ramani, G.; Chan, S.Y.; Waxman, A.B. Rationale and Design of the Ranolazine PH-RV Study: A Multicentred Randomised and Placebo-Controlled Study of Ranolazine to Improve RV Function in Patients with Non-Group 2 Pulmonary Hypertension. *Open Heart* **2018**, *5*, e000736, doi:10.1136/openhrt-2017-000736.
26. Han, Y.; Forfia, P.; Vaidya, A.; Mazurek, J.A.; Park, M.H.; Ramani, G.; Chan, S.Y.; Waxman, A.B. Ranolazine Improves Right Ventricular Function in Patients With Precapillary Pulmonary Hypertension: Results From a Double-Blind, Randomized, Placebo-Controlled Trial. *J. Card. Fail.* **2021**, *27*, 253–257, doi:10.1016/j.cardfail.2020.10.006.
27. Arnold, W.D.; Kline, D.; Sanderson, A.; Hawash, A.A.; Bartlett, A.; Novak, K.R.; Rich, M.M.; Kissel, J.T. Open-Label Trial of Ranolazine for the Treatment of Myotonia Congenita. *Neurology* **2017**, *89*, 710–713, doi:10.1212/WNL.0000000000004229.
28. Lorusso, S.; Kline, D.; Bartlett, A.; Freimer, M.; Agriesti, J.; Hawash, A.A.; Rich, M.M.; Kissel, J.T.; David Arnold, W. Open-Label Trial of Ranolazine for the Treatment of Paramyotonia Congenita. *Muscle Nerve* **2019**, *59*, 240–243, doi:10.1002/mus.26372.
29. Minotti, G.; Menna, P.; Calabrese, V.; Greco, C.; Armento, G.; Annibali, O.; Marchesi, F.; Salvatorelli, E.; Reggiardo, G. Pharmacology of Ranolazine versus Common Cardiovascular Drugs in Patients with Early Diastolic Dysfunction Induced by Anthracyclines or Nonanthracycline Chemotherapeutics: A Phase 2b Minitrial. *J. Pharmacol. Exp. Ther.* **2019**, *370*, 197–205, doi:10.1124/jpet.119.258178.
